# Apelin-13 Protects Dopaminergic Neurons against Rotenone—Induced Neurotoxicity through the AMPK/mTOR/ULK-1 Mediated Autophagy Activation

**DOI:** 10.3390/ijms21218376

**Published:** 2020-11-08

**Authors:** Peng Chen, Youcui Wang, Leilei Chen, Ning Song, Junxia Xie

**Affiliations:** 1 Institute of Brain Science and Disease, Shandong Provincial Key Laboratory of Pathogenesis and Prevention of Neurological Disorders, Shandong Provincial Collaborative Innovation Center for Neurodegenerative Disorders, Qingdao University, Qingdao 266071, China; chenpengqingda@163.com (P.C.); wangyoucui1987@163.com (Y.W.); hanbing_leilei@163.com (L.C.); 2 Department of Physiology, College of Basic Medicine, Shandong First Medical University & Shandong Academy of Medical Sciences, Jinan 250000, China

**Keywords:** Parkinson’s disease, neuroprotection, apelin-13, autophagy, AMPK/mTOR/ULK-1 signaling

## Abstract

Parkinson’s disease (PD) is characterized by the progressive loss of dopaminergic neurons in the substantia nigra pars compacta. Several brain–gut peptides are able to exert neuroprotective effects on the nigrostriatal dopaminergic system. Apelin-13 is a neuropeptide, conveying potential neuroprotective activities. However, whether, and how, apelin-13 could antagonize rotenone-induced neurotoxicity has not yet been elucidated. In the present study, rotenone-treated SH-SY5Y cells and rats were used to clarify whether apelin-13 has protective effects on dopaminergic neurons, both in vivo and in vitro. The results showed that apelin-13 could protect SH-SY5Y cells from rotenone-induced injury and apoptosis. Apelin-13 was able to activate autophagy, and restore rotenone induced autophagy impairment in SH-SY5Y cells, which could be blocked by the autophagy inhibitor 3-Methyladenine. Apelin-13 activated AMPK/mTOR/ULK-1 signaling, AMPKα inhibitor compound C, as well as apelin receptor blockage via siRNA, which could block apelin-13-induced signaling activation, autophagy activation, and protective effects, in rotenone-treated SH-SY5Y cells. These results indicated that apelin-13 exerted neuroprotective properties against rotenone by stimulating AMPK/mTOR/ULK-1 signaling-mediated autophagy via the apelin receptor. We also observed that intracerebroventricular injection of apelin-13 could alleviate nigrostriatal dopaminergic neuron degeneration in rotenone-treated rats. Our findings provide new insights into the mechanism by which apelin-13 might attenuate neurotoxicity in PD.

## 1. Introduction

Parkinson’s disease (PD) is the second common neurodegenerative disease, and is characterized by the progressive loss of dopaminergic neurons in the substantia nigra pars compacta (SNpc) and the presence of Lewy bodies composed of aggregated α-synuclein [1,2]. It has been identified that oxidative stress, mitochondrial dysfunction, and an impaired protein degradation system are involved in the onset and progression of PD [3]. There are no satisfactory strategies that slow down the neurodegeneration of dopaminergic neurons in PD. Current therapy strategies, such as dopamine agonists and L-3,4-dihydroxyphenylalanine (L-DOPA), are symptomatic treatments for PD, but they fail to render disease-modifying or continual impacts [4]. Several brain-gut peptides, such as ghrelin, nesfatin-1, neurotensin, neuropeptide Y (NPY), and glucagon-like peptide-1, are widely expressed in the mammalian central nervous system, and have a close relationship with the central dopaminergic system [5,6,7,8,9,10,11]. We previously reported that ghrelin and nesfatin-1 were able to antagonize 1-methyl-4-phenylpyridinium ion-induced cytotoxicity in MES23.5 dopaminergic cells by the anti-apoptotic C-Raf-ERK1/2 pathway, anti-oxidative stress pathway, and inhibiting the translocation of NF-κB [8,11].

Apelin is a kind of brain–gut peptide that serves as an orphan G protein-coupled apelin receptor (putative receptor protein related to the angiotensin receptor ATl, APJ) ligand. Apelin is derived from stolid abdominal tissues, and sustains cleaved form apelin-13, apelin-17, and apelin-36 [12], all of which can bind to receptor APJ, and then activate following second messenger signaling cascades [13]. Apelin-13 serves as the most effective type of apelin in competing to interact with the APJ [14]. The distribution of apelin-13 in the hypothalamus, substantia nigra (SN), striatum (Str), cerebellum, hippocampus, hypothalamus, and amygdala, implies that apelin-13 may display crucial and broad functions in pathophysiological and physiological processes [15]. Recent reports showed that apelin-13 protects SH-SY5Y dopaminergic cells from 6-hydroxydopamine-induced neurotoxicity by its antioxidant and anti-apoptotic properties [16]. In vivo, apelin-13 protects dopaminergic neurons in MPTP-induced PD mice through inhibiting endoplasmic reticulum stress and promoting autophagy [17]. Rotenone is a lipid-soluble environmental toxin in daily life. Rotenone is able to enter the human digestive tract in the form of pesticide residues, and pass through the blood–brain barrier [18]. Whether apelin-13 could be neuroprotective in rotenone models has not yet been investigated.

Autophagy, as the process of cellular self-digestion, is a pathway involving degradation of organelles and protein, and containing an extraordinary quantity of links to physiological processes and human diseases [19]. It has been well-acknowledged that autophagy is associated with PD [20]. The autophagic pathway had protective effects on dopaminergic neurons by removing accumulated α-synuclein in SNpc, in most PD-animal models, eventually promoting cell survival [21,22]. Accordingly, inducers of autophagy have been recognized as neuroprotectors for PD. The autophagy inducer, rapamycin, serves as a neuroprotective candidate in PD models, by intensifying autophagy to diminish misfolded protein [23]. Carbamazepine and valproic acid, serving as potential autophagy enhancers, attenuated rotenone-induced toxicity in SH-SY5Y cells [24]. Increasing evidence indicates that apelin-13 is able to regulate autophagy in SH-SY5Y cell, rat cardiomyocytes, lung adenocarcinoma cell, foam cell, and rat cerebral etc. [25,26,27,28]. AMPK/mTOR/ULK1 signaling is involved in the modulation of autophagy in PD [29]. However, whether apelin-13 could induce autophagy via AMPK/mTOR/ULK1 signaling remains elusive.

In the present study, rotenone was employed to establish PD models both in vitro, and in vivo, to mimic the environmental insults in PD. We aimed to explore the protective effects of apelin-13 in rotenone-treated SH-SY5Y cells, and in rats, and to further investigate the neuroprotection of apelin-13, related to AMPK/mTOR/ULK1 signaling-mediated autophagy.

## 2. Results

### 2.1. Apelin-13 Antagonized Rotenone-Induced Cell Injury in SH-SY5Y Cells

**Figure 1:**
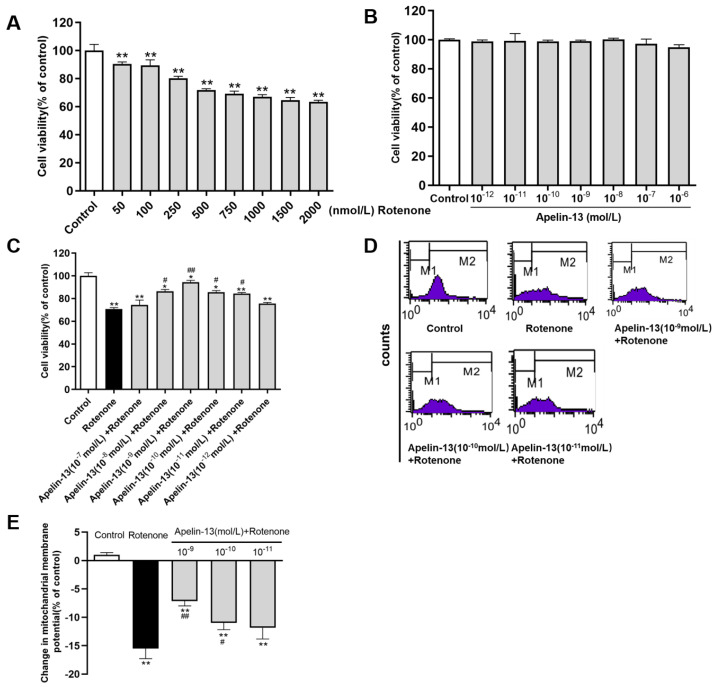
Cell viability and mitochondrial evaluation in SH-SY5Y cells with rotenone and apelin-13 treatment. (**A**–**C**) SH-SY5Y cells were treated with rotenone (50 nM ~ 2000 nM) or apelin-13 (10^−12^ mol/L ~ 10^−6^ mol/L), or exposed to rotenone (500 nM) with or without the pretreatment apelin-13 (10^−12^ mol/L ~ 10^−7^ mol/L) for 24 h. Cell viability was tested by MTT assays and data were presented as percentage of control. Restoration of viability was found when cells were pre-incubated with apelin-13 (10^−11^ mol/L ~ 10^−8^ mol/L). (**D**,**E**) Fluorometric assays of mitochondrial membrane potential (ΔΨm) and statistical analysis. Rotenone treatment for 24 h resulted in a ΔΨm decrease, which was abolished by pre-incubation with apelin-13 (10^−10^ mol/L and 10^−9^ mol/L). Data are presented as mean ± SEM of three replications. * *p* < 0.05; ** *p* < 0.01 compared with the control; ^#^ *p* < 0.05; ^##^ *p* < 0.01 compared with the rotenone group.

The protective effects of apelin-13 against rotenone induced neurotoxicity were investigated in SH-SY5Y cells. The SH-SY5Y cells were treated with rotenone for 24 h. The results showed that the cell viability was decreased in SH-SY5Y cells treated with rotenone, from 50 nM to 2000 nM, compared with the control (Figure 1A). Then the dose of 500 nM of rotenone was used in the following analysis. Apelin-13 (10^−12^ mol/L ~ 10^−6^ mol/L) treatment did not affect the cell viability of SH-SY5Y cells (Figure 1B). Apelin-13 (10^−11^ mol/L ~ 10^−8^ mol/L) pretreatment for 24 h was able to restore cell viability in rotenone-treated SH-SY5Y cells (Figure 1C). The maximum protection was achieved at the concentration of 10^−9^ mol/L apelin-13. Similarly, rotenone treatment resulted in a mitochondrial membrane potential (ΔΨm) decrease, assessed by flow cytometry, after a 24 h exposure. Pre-incubation with 10^−9^ mol/L apelin-13 exerted maximal protective effects (Figure 1D,E).

### 2.2. Apelin-13 Inhibited Rotenone-Induced Apoptosis in SH-SY5Y Cells

**Figure 2:**
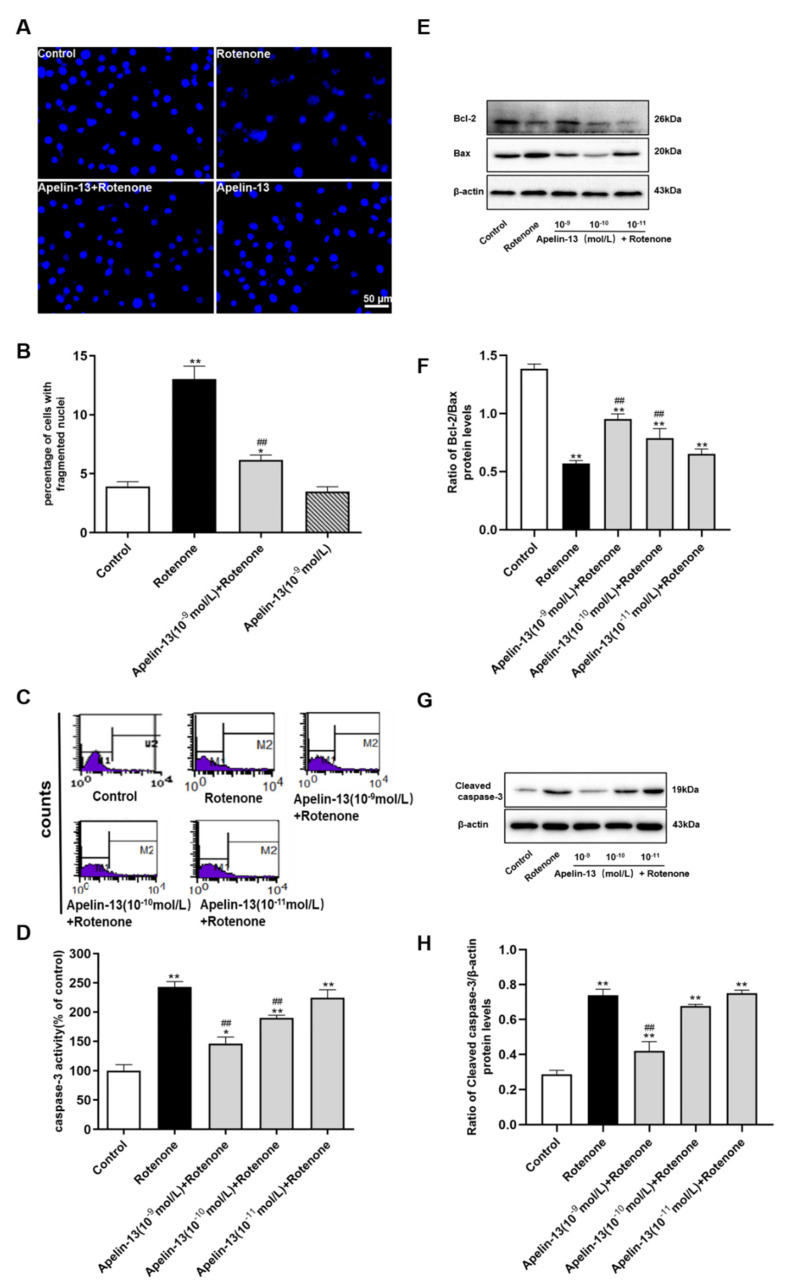
Effects of apelin-13 on rotenone-induced apoptosis in SH-SY5Y cells. (**A**) The cells with fragmented nuclei were measured by Hochest 33258 staining. Rotenone-induced nuclear condensation was attenuated by apelin-13 pretreatment. Bar = 50 μm. (**B**) Statistical analysis. * *p* < 0.05; ** *p* < 0.01 compared with the control; ^##^ *p* < 0.01 compared with the rotenone group. (**C**) Fluorometric assay of caspase-3 activity in cells treated with vehicle or apelin-13 (10^−11^ mol/L ~ 10^−9^ mol/L) prior to rotenone treatment for 24 h. Caspase-3 activity was increased in cells with rotenone treatment; however, this effect was attenuated by apelin-13 pretreatment. (**D**) Statistical analysis. Data are presented as mean ± SEM of three replications. * *p* < 0.05; ** *p* < 0.01 compared with Control; ^##^ *p* < 0.01 compared with the rotenone group. (**E**–**H**) SH-SY5Y cells were treated with rotenone or pre-treated with apelin-13 (10^−11^ mol/L ~ 10^−9^ mol/L) prior to rotenone treatment for 24 h. Western blot was applied to detect the expression of Bcl-2, Bax, cleaved caspase-3. β-actin was used as a loading control. Apelin-13 pretreatment decreased the expression of Bax and cleaved caspase-3, while increased the expression of Bcl-2 in the rotenone-treated SH-SY5Y cells. Data are presented as mean ± SEM of three replications. ** *p* < 0.01 compared with the control; ^##^ *p* < 0.01 compared with the rotenone group.

We then evaluated whether apelin-13 could inhibit rotenone induced apoptosis. Hoechst 33258 staining showed that cells with round and large sized nuclei appeared with regular contours in the control and apelin-13 alone groups. However, rotenone treatment caused condensed chromatin and bright apoptotic nuclei in SY-SY5Y cells, which was significantly attenuated by pre-treatment with apelin-13 (10^−9^ mol/L) (Figure 2A,B). Rotenone caused a dramatic elevation of caspase-3 activity in SH-SY5Y cells, apelin-13 (10^−10^ mol/L and 10^−9^ mol/L) inhibited the rotenone-induced caspase-3 activity, while apelin-13 (10^−11^ mol/L) had no effects (Figure 2C,D). We also observed rotenone induced up-regulation of pro-apoptotic factor Bax, and down-regulation of anti-apoptotic protein Bcl-2, thus a decrease in ratio of Bcl-2/Bax. Apelin-13 (10^−10^ mol/L and 10^−9^ mol/L) restored the ratio of Bcl-2/Bax in rotenone-treated SH-SY5Y cells, while apelin-13 (10^−11^ mol/L) had no effects (Figure 2E,F). The anti-apoptotic effects of apelin-13 were further evidenced by the inhibition of cleaved caspase-3 up-regulation induced by rotenone; this effect was only observed in cells with 10^−9^ mol/L apelin-13 pretreatment, rather than 10^−11^ mol/L and 10^−10^ mol/L apelin-13 pretreatment (Figure 2G,H).

### 2.3. Apelin-13 Induced Autophagy and Restored Rotenone Induced Autophagy Impairment in SH-SY5Y Cells

**Figure 3:**
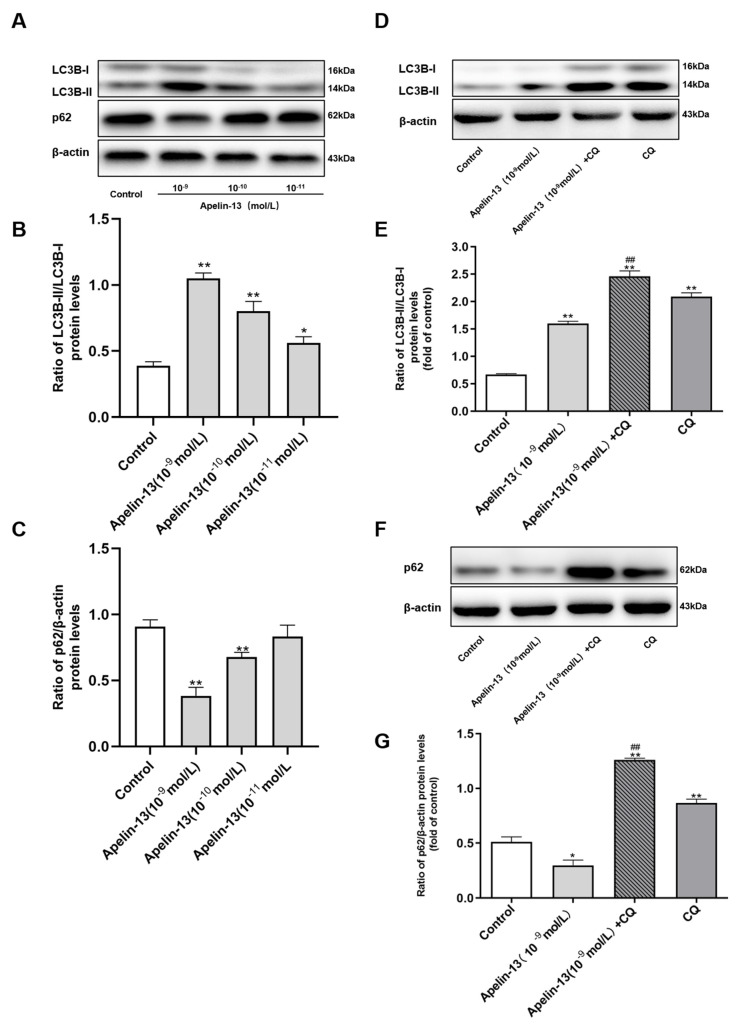
Autophagy activation in SH-SY5Y cells with apelin-13 treatment. (**A**–**C**) SH-SY5Y cells were treated with apelin-13 (10^−11^ mol/L ~ 10^−9^ mol/L) for 24 h. Western blot was applied to detect the expression of light chain 3B (LC3B)-II/LC3B-I and p62. The ratio of LC3B-II/LC3B-I was increased dose-dependently with apelin-13 (10^−10^ mol/L ~ 10^−9^ mol/L) treatment, while the expression of p62 was reduced as compared with the control. (**D**–**G**) SH-SY5Y cells were treated with apelin-13 or co-treated with apelin-13 and CQ (10 μM). The ratio of LC3B-II/LC3B-I and the expression of p62 were increased in the presence of CQ, and further increased with apelin-13 and CQ in SH-SY5Y cells. Data are presented as mean ± SEM of three replications. * *p* < 0.05; ** *p* < 0.01 compared with the control; ^##^ *p* < 0.01 compared with the apelin-13 group.

We further explored the effects of apelin-13 on autophagy in SH-SY5Y cells. Western blot revealed that the ratio of light chain 3B (LC3B)-II/LC3B-I was increased dose-dependently with apelin-13 (10^−11^ mol/L ~ 10^−9^ mol/L) treatment, while apelin-13 (10−^10^ mol/L and 10^−9^ mol/L) reduced the expression of the autophagic adaptor protein p62, compared with the control (Figure 3A–C), indicating that apelin-13 induced autophagy in SH-SY5Y cells. To show that an increased generation of new autophagosomes, rather than a blockade of their lysosomal degradation, was responsible for the increased LC3B-II/LC3B-I ratio, cells were pre-treated with 30 μM chloroquine (CQ), a known inhibitor of autophagosome-lysosome-fusion for 6 h prior to apelin-13. CQ alone induced an increased ratio of LC3B-II/LC3B-I, and increased expression of p62. CQ pre-treatment, prior to apelin-13, further increased the ratio of LC3B-II/LC3B-I, meanwhile, reversing the reduction of p62 in SH-SY5Y cells (Figure 3D–G).

**Figure 4:**
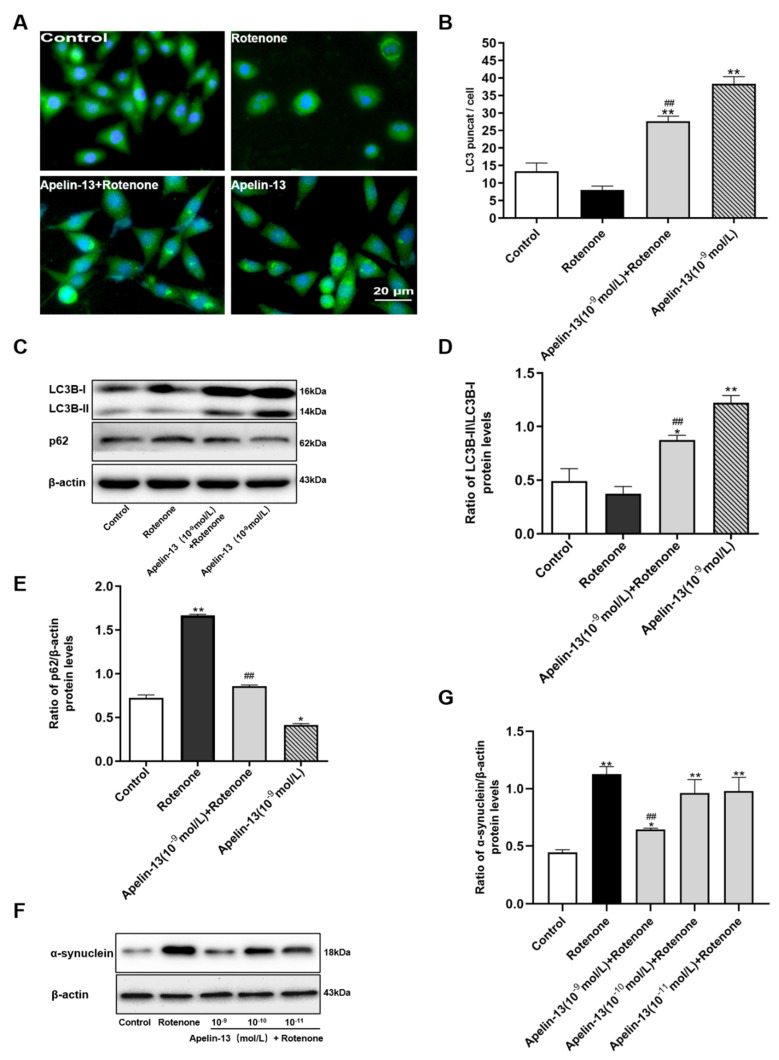
Effects of apelin-13 on rotenone-induced autophagy impairment in SH-SY5Y cells. (**A**) Fluorescent images were captured and processed. Cells in the control and rotenone groups exhibited a diffuse distribution of LC3BⅡ. The number of LC3BⅡpuncta increased in the presence of apelin-13, with or without rotenone treatment. Bar = 20 μm. (**B**) Statistical analysis of LC3 puncta/cell. Data are presented as mean ± SEM of three replications. ** *p* < 0.01 compared with the control; ^##^ *p* < 0.01 compared with the rotenone group. (**C**–**E**) Western blot was applied to detect the levels of LC3B-II/LC3B-I and p62. Apelin-13 rescued the ratio of LC3B-II/LC3B-I and blocked the expression of p62 in the rotenone-treated SH-SY5Y cells. (**F**,**G**) Western blot was applied to detect the expression of α-synuclein. Rotenone treatment led to a dramatic increase in α-synuclein levels in SH-SY5Y cells; however, apelin-13 pretreatment significantly reduced this increase. Data are presented as mean ± SEM of three replications. * *p* < 0.05; ** *p* < 0.01 compared with the control; ^##^ *p* < 0.01 compared with the rotenone group.

To further determine whether apelin-13 could induce autophagy in rotenone treated cells, we observed the fluorescent punctuate distribution of LC3B by immunofluorescence staining with anti-LC3B antibody. The results showed that apelin-13 (10^−9^ mol/L) treated cells exhibited increased punctuate patterns of LC3B fluorescence distribution in the cytoplasm of cells, whereas, diffuse distribution of LC3B appeared in the absence of apelin-13. SH-SY5Y cells with apelin-13 pre-treatment prior to rotenone also showed a significant increase versus cells treated with rotenone (Figure 4A). In addition, p62 was accumulated in rotenone-treated cells, apelin-13 blocked the expression of p62 in the rotenone-treated SH-SY5Y cells, as well as increasing the ratio of LC3B-II/LC3B-I (Figure 4B–D). Then, we observed the effects of apelin-13 on the protein expression of α-synuclein in SH-SY5Y cells. The results showed that the expression levels of α-synuclein were up-regulated in cells with rotenone treatment, which could be blocked by 10^−9^ mol/L apelin-13 pretreatment. Low concentration of apelin-13 (10^−11^ mol/L and 10^−10^ mol/L) did not show any effects (Figure 4E,F). Taken together, these data suggest that apelin-13 is able to induce autophagy in SH-SY5Y cells.

### 2.4. Autophagy Activation Contributed to the Protective Effects of Apelin-13 in Rotenone Treated SH-SY5Y Cells

**Figure 5:**
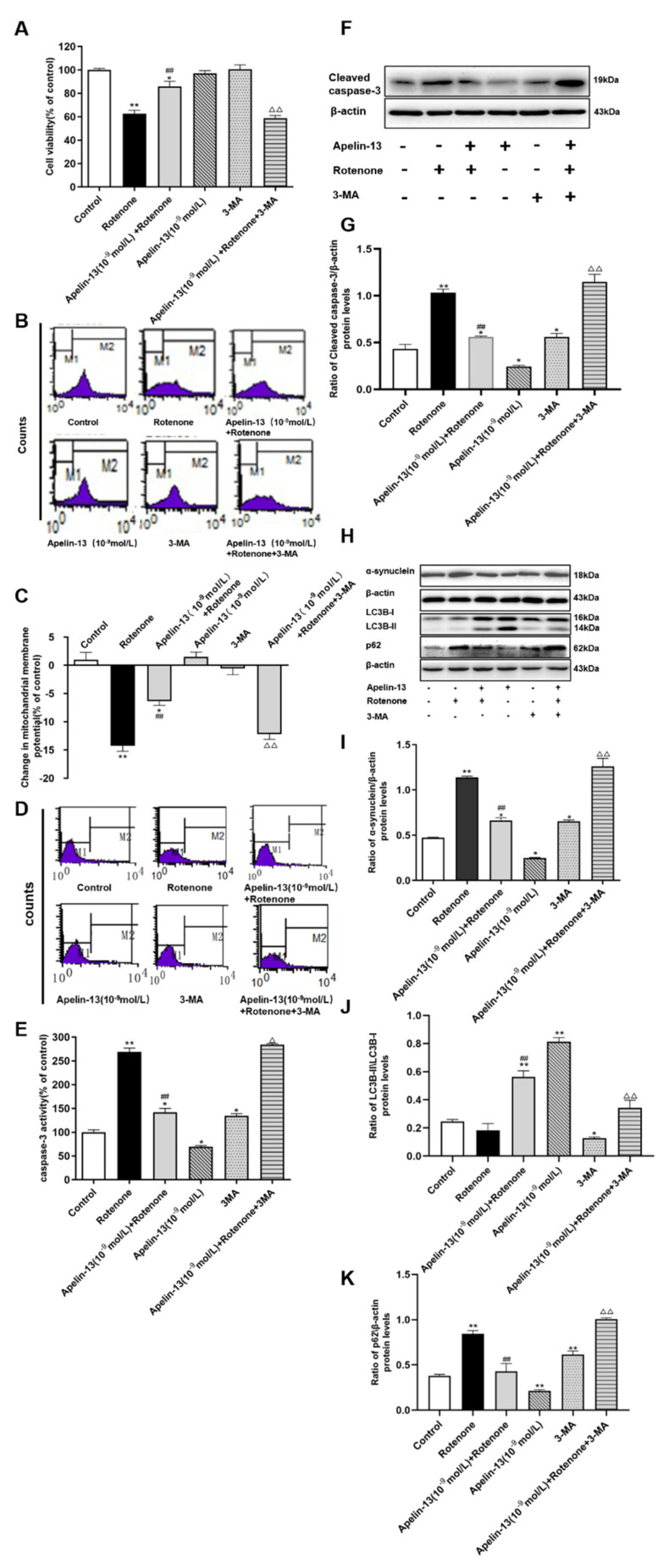
Effects of 3-MA on the neuroprotective effects of apelin-13 in SH-SY5Y cells. SH-SY5Y cells were exposed to rotenone with or without apelin-13 pretreatment, or with 3-MA pretreatment prior to rotenone and apelin-13. (**A**) MTT assay indicated that 3-MA had no effects of cell viability. Apelin-13 restored cell viability in rotenone-treated cells, which was blocked by 3-MA. (**B**,**C**) Fluorometric assays of ΔΨm and statistical analysis. Apelin-13 antagonized rotenone-induced reduction in △ΨM, and pre-incubation with 3-MA abolished these changes. (**D**,**E**) Fluorometric assay of caspase-3 activity and statistical analysis. Apelin-13 could antagonize rotenone-induced caspase-3 activation, and pre-incubation with 3-MA abolished the activation. (**F**,**G**) Western blot was applied to detect the expression of cleaved caspase-3. Apelin-13 antagonize rotenone-induced the increase of cleaved caspase-3 protein levels, and pre-incubation with 3-MA abolished these changes. (**H**–**K**) Western blot was applied to detect the expression of α-synuclein, LC3B-II/LC3B-I, and p62. Apelin-13 enhanced the ratio of LC3B-II/LC3B-I, and reduced the expression of p62 and α-synuclein in the rotenone-treated cells; 3-MA pretreatment blocked these effects. Data are presented as mean ± SEM of three replications. * *p* < 0.05; ** *p* < 0.01 compared with the control; ^##^ *p* < 0.01 compared with the rotenone group; ^△^ *p* < 0.05; ^△△^ *p* < 0.01 compared with the Apelin-13+Rotenone group.

We further investigated whether apelin-13 exerted its protective effects on SH-SY5Y cells from rotenone-induced injury by the stimulation of autophagy. MTT assays revealed that the treatment of apelin-13 (10^−9^ mol/L) rescued the cell viability reduced by rotenone in SH-SY5Y cells, while the autophagy inhibitor 3-MA pretreatment for 3 h, could inhibit this effect. 3-MA alone did not show any significant effect on cell viability (Figure 5A). 3-MA also had no effects on ΔΨm, however, it induced a small increase of both, caspase-3 activity, and cleaved caspase-3 protein levels. Consistent with data in Figure 2, apelin-13 pretreatment inhibited the caspase-3 activity and cleaved caspase-3 induced by rotenone; 3-MA was able to block the protective effects of apelin-13 (Figure 5B–G). As we expected, 3-MA alone resulted in a low ratio of LC3B-II/LC3B-I and accumulated p62 and α-synuclein in SH-SY5Y cells, consistent with its property as an autophagy inhibitor. Apelin-13 induced autophagy in rotenone treated cells, as indicated by a higher ratio of LC3B-II/LC3B-I, and less p62 and α-synuclein, and was blocked with 3-MA pretreatment (Figure 5H–K). Taken together, these facts indicate that apelin-13 can protect SH-SY5Y cells from rotenone-induced injury and apoptosis by activating autophagy.

### 2.5. Apelin-13 Is Able to Modulate the AMPK-/mTOR-/ULK1 Signaling in SH-SY5Y Cells

**Figure 6:**
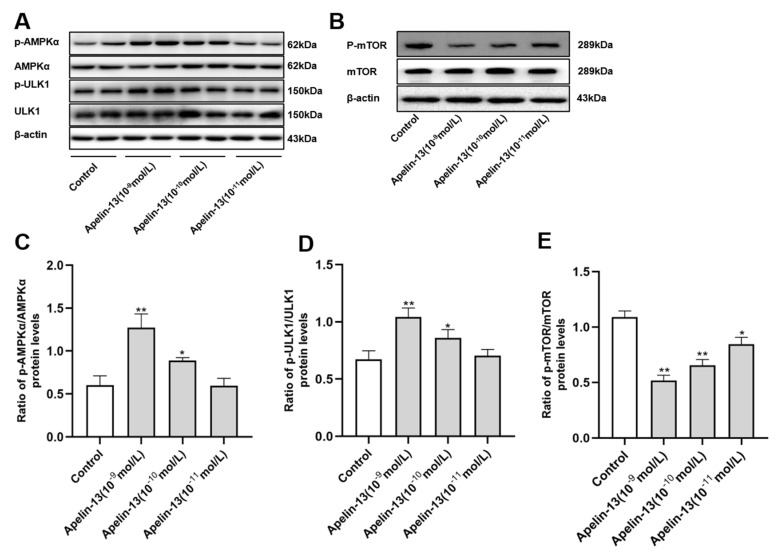
The AMPK/mTOR/ULK1 signaling pathway activation in SH-SY5Y cells with apelin-13 treatment. SH-SY5Y cells were treated with apelin-13 (10^−11^ mol/L ~ 10^−9^ mol/L) at the indicated dose. (**A**–**E**) Western blot was applied to detect the phosphorylated and total AMPKα, mTOR, and ULK1. The ratios of p-AMPK/AMPK and p-ULK1/ULK1 were increased dose-dependently; the ratio of p-mTOR/mTOR was decreased dose-dependently in cells with apelin-13 treatment, when compared with the control group. Data are presented as mean ± SEM of three replications. * *p* < 0.05; ** *p* < 0.01 compared with the control.

AMPK/mTOR/ULK1 signaling was identified as modulating autophagy in the progression of PD [30]. Accordingly, we tried to explore whether AMPK/mTOR/ULK1 signaling was involved in apelin-13-induced autophagy in SH-SY5Y cells. Apelin-13 (10^−9^ mol/L and 10^−10^ mol/L) was able to enhance the phosphorylation of AMPKα and ULK1, although apelin-13 (10^−11^ mol/L) had no effect on the phosphorylation of AMPKα and ULK1 (Figure 6A,C,D); 10^−11^ mol/L~10^−9^ mol/L apelin-13 dose-dependently reduced the phosphorylation of mTOR in the cells (Figure 6B,E).

### 2.6. AMPK/mTOR/ULK1 Signaling Contributed to the Protective Effects of Apelin-13 in Rotenone Treated SH-SY5Y Cells

**Figure 7:**
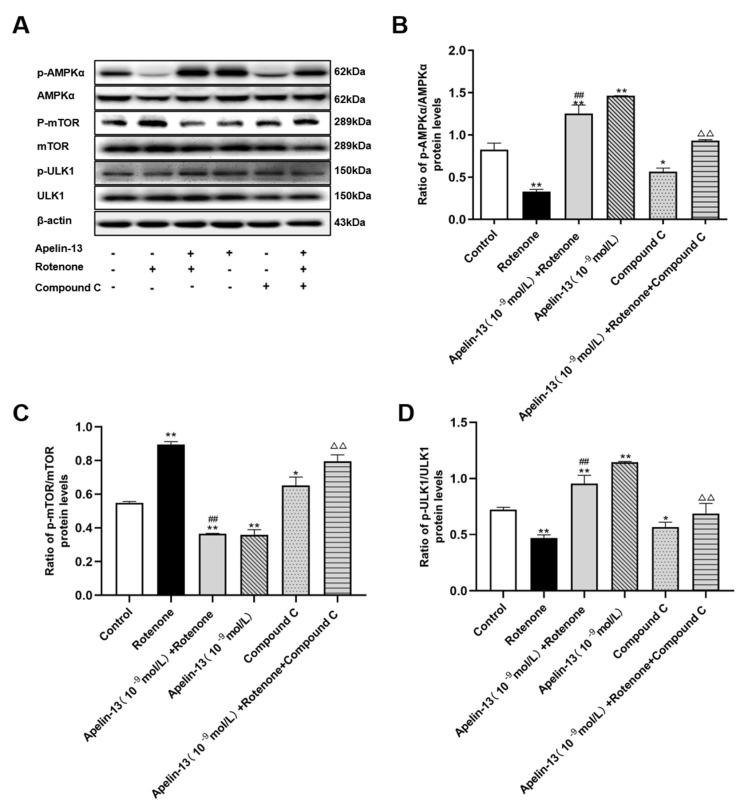
Effects of compound C on the AMPK/mTOR/ULK1 signaling pathway in SH-SY5Y cells with rotenone and apelin-13 treatment. SH-SY5Y cells were exposed to rotenone with or without apelin-13, or with compound C pretreatment prior to rotenone and apelin-13. (**A**–**D**) Western blot was applied to detect the phosphorylated and total AMPKα, mTOR, and ULK1. Apelin-13 antagonized rotenone induced an increase in the ratios of p-AMPK/AMPK and p-ULK1/ULK1, and rotenone induced a reduction in the ratio of p-mTOR/mTOR; compound C pretreatment blocked these effects. Data are presented as mean ± SEM of three replications. * *p* < 0.05; ** p < 0.01 compared with the control; ^##^ *p* < 0.01 compared with the rotenone group; ^△△^ *p* < 0.01 compared with the Apelin-13+Rotenone group.

In order to determine whether AMPK/mTOR/ULK1 signaling is involved in the protection and autophagy activation of apelin-13 in rotenone-treated SH-SY5Y cells, an AMPKα inhibitor, compound C, was used 30 min before apelin-13 treatment. The results showed rotenone induced the phosphorylation of mTOR and inhibited the phosphorylation of AMPKα and ULK1. Consistent with the effects of apelin-13 in normal SH-SY5Y cells, apelin-13 was able to reverse the phosphorylation status of AMPK/mTOR/ULK1 signaling in rotenone treated cells, that is, more phosphorylated AMPKα and ULK1, and less phosphorylated mTOR. Compound C alone exerted similar effects to rotenone; meanwhile, it efficiently blocked the effects of apelin-13 on the AMPK/mTOR/ULK1 signaling pathway (Figure 7A–D).

**Figure 8:**
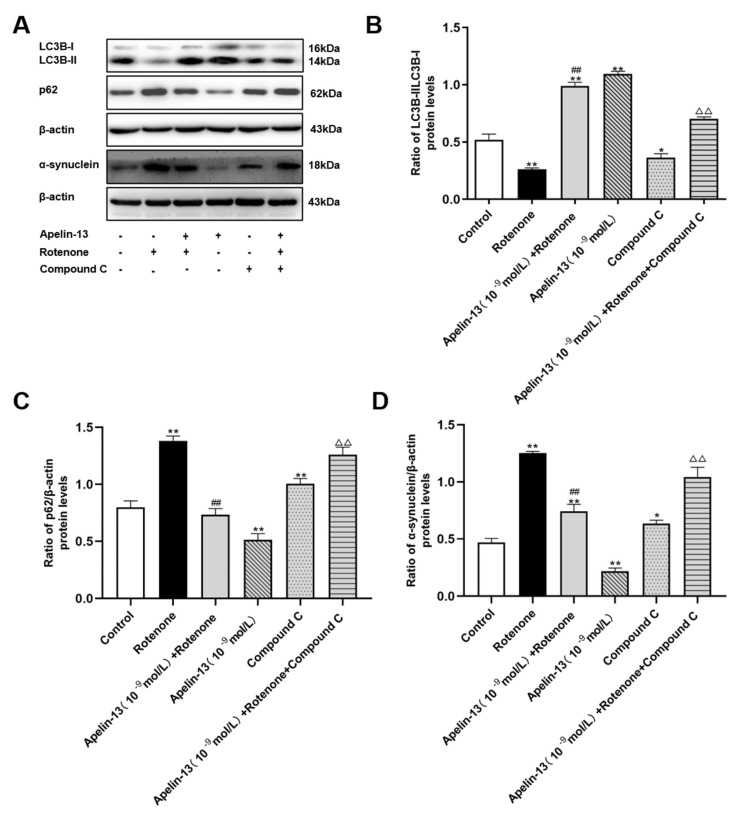
Effects of compound C on autophagy in SH-SY5Y cells with rotenone and apelin-13 treatments. (**A**–**D**) Western blot was applied to detect the expression of LC3B-II/LC3B-I, p62 and α-synuclein in the cells. Apelin-13 enhanced the ratio of LC3B-II/LC3B-I, and reduce the expression of p62 and α-synuclein in the rotenone-treated cells; these effects were blocked by compound C. Data are presented as mean ± SEM of three replications. * *p* < 0.05; ** p < 0.01 compared with the control; ^##^ *p* < 0.01 compared with the rotenone group; ^△△^ *p* < 0.01 compared with the Apelin-13+Rotenone group.

We then explored whether apelin-13 induces autophagy by stimulating AMPK/mTOR/ULK1 signaling in SH-SY5Y cells. Compound C alone induced a low ratio of LC3B-II/LC3B-I, and accumulated p62 and α-synuclein in SH-SY5Y cells, indicating its certain autophagy inhibitory properties. Apelin-13 enhanced the ratio of LC3B-II/LC3B-I, and reduced the expression of p62 and α-synuclein in rotenone-treated cells, and these effects were blocked by compound C (Figure 8A–D).

**Figure 9:**
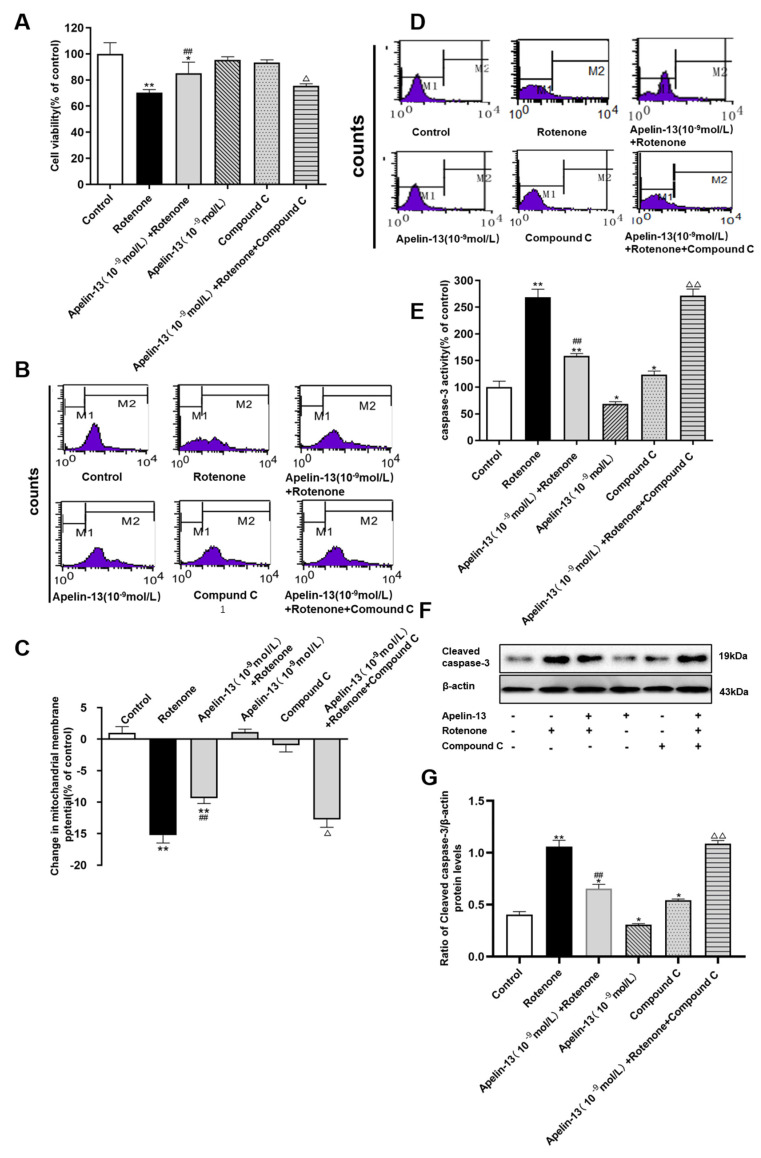
Effects of compound C on the neuroprotective effects of apelin-13 in SH-SY5Y cells. (**A**) The cell viability was tested by MTT assays, and is presented as percentage of control. Apelin-13 rescued rotenone-induced cell death, and this effect was prevented by compound C. (**B**,**C**) Fluorometric assays of ΔΨm and statistical analysis. Compound C pretreatment abolished the protective effects of apelin-13 on ΔΨm. (**D**,**E**) Fluorometric assay of caspase-3 activity and statistical analysis. Apelin-13 antagonize rotenone-induced caspase-3 activation, and pre-incubation with compound C abolished these changes. (**F**,**G**) Western blot was applied to detect the expression of cleaved caspase-3. Apelin-13 antagonized rotenone-induced the increase in cleaved caspase-3 protein levels, and compound C pretreatment abolished these changes. Data are presented as mean ± SEM of three replications. * *p* < 0.05; ** p < 0.01 compared with the control; ^##^ *p* < 0.01 compared with the rotenone group; ^△^ *p* < 0.05; ^△△^ *p* < 0.01 compared with the Apelin-13+Rotenone group.

Next we further explored whether apelin-13 exerted its protective effect by the stimulation of AMPK/mTOR/ULK1 signaling in SH-SY5Y cells. As shown in Figure 9A, compound C, alone, did not show any significant effect on cell viability. Compound C also had no effects on ΔΨm, however, it induced a small increase of both caspase-3 activity and cleaved caspase-3 protein levels (Figure 9B–G). The cell viability and ΔΨm were restored by apelin-13 in rotenone-treated SH-SY5Y cells, both of which were abolished by compound C pretreatment (Figure 9A–C). Apelin-13 antagonize rotenone-induced caspase-3 activation and cleaved caspase-3 protein expression, and pre-incubation with compound C, abolished these protective effects. (Figure 9D–G).

### 2.7. APJ Contributed to the Protective Effects of Apelin-13 in Rotenone Treated SH-SY5Y Cells

**Figure 10:**
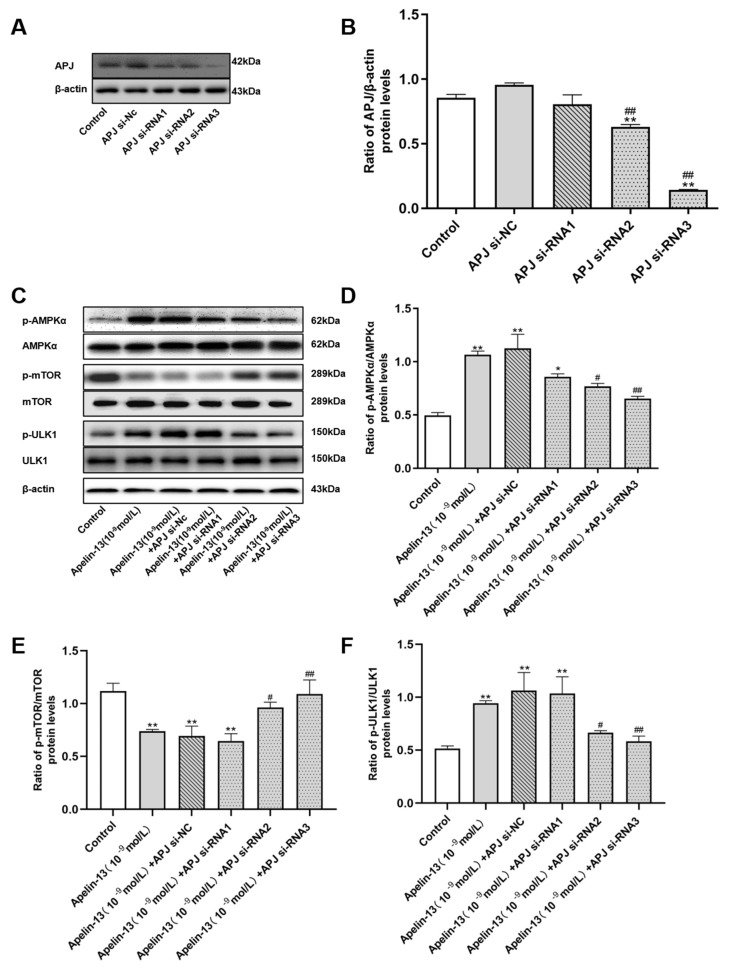
Effects of apelin receptor (APJ) siRNA on the AMPK/mTOR/ULK1 signaling pathway in SH-SY5Y cells with apelin-13 treatment. (**A**,**B**) SH-SY5Y cells were transfected with APJ siRNA. Western blot was applied to detect the APJ protein expression. The expression of APJ protein was inhibited by siRNA. (**C**–**F**) SH-SY5Y cells were treated with apelin-13 or pre-treated with APJ siRNA prior to apelin-13. Western blot was applied to detect the phosphorylated and total AMPKα, mTOR, and ULK1. Phosphorylation of AMPKα and ULK1 were attenuated in apelin-13 treated cell with APJ siRNA, while phosphorylation of mTOR was increased. Data are presented as mean ± SEM of three replications. * *p* < 0.05; ** *p* < 0.01 compared with the control; ^#^ *p* < 0.05; ^##^ *p* < 0.01 compared with the apelin-13 group.

The efficiency of APJ depletion by APJ siRNA was validated in the cells (Figure 10A,B). The results show that APJ siRNA2 and APJ siRNA3 could differently reduce the expression of APJ by 26.28% and 83.49%, compared with the siRNA-Nc group. SH-SY5Y cells with APJ siRNA transfection were treated with apelin-13 for 24 h. Phosphorylation of AMPKα and ULK1 was increased, while the phosphorylation of mTOR was decreased in SH-SY5Y cells with apelin-13 treatments; APJ siRNA2 and APJ siRNA3 were able to block these effects (Figure 10C–F).

**Figure 11:**
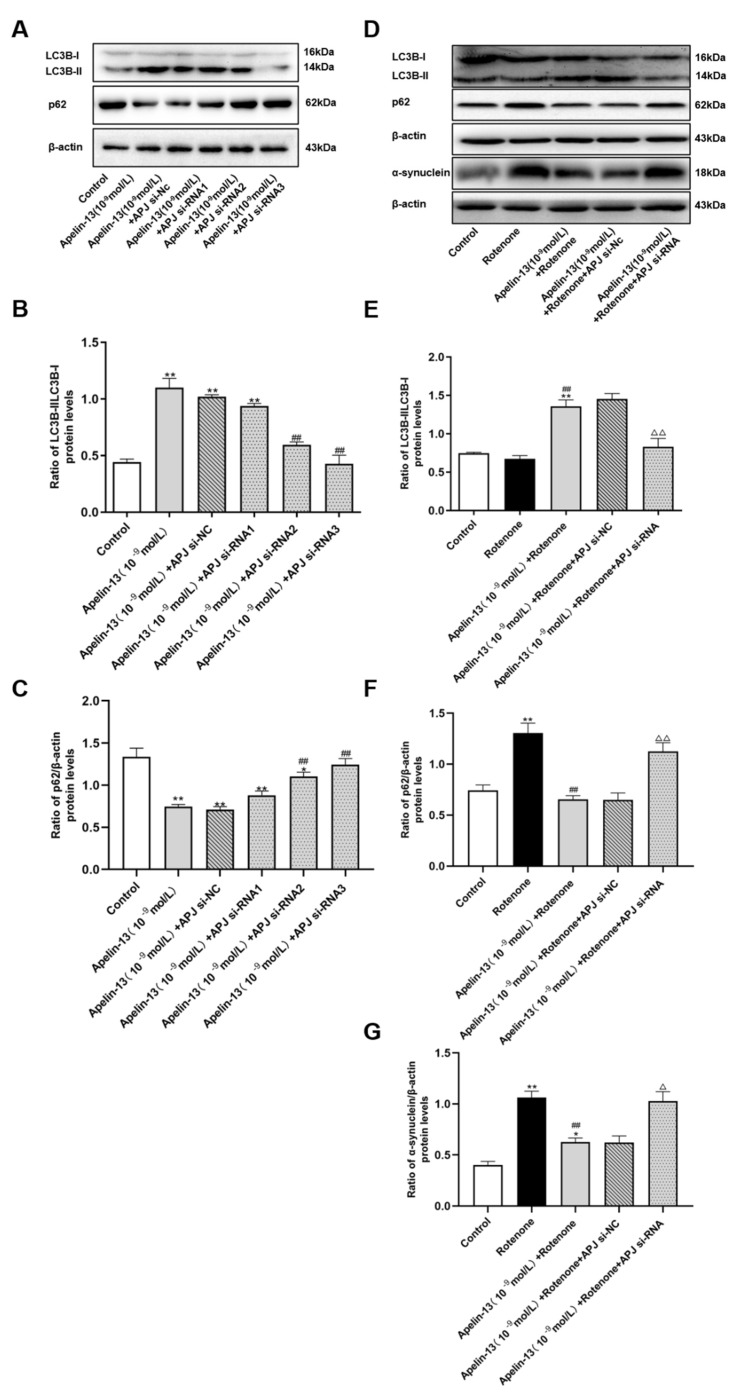
Effects of APJ siRNA on autophagy in SH-SY5Y cells with rotenone and apelin-13 treatment. (**A**–**C**) SH-SY5Y cells were treated with apelin-13 or co-treated with apelin-13 and APJ siRNA. Western blot was applied to detect the phosphorylation of LC3B-II/LC3B-I and p62. APJ siRNA abolished the increased LC3B-II/LC3B-I ratio induced by apelin-13, and restored p62 levels in the SH-SY5Y cells. Data are presented as mean ± SEM of three replications. * *p* < 0.05; ** *p* < 0.01 compared with the control; ^#^ *p* < 0.05; ^##^ *p* < 0.01 compared with the apelin-13 group. (**D**–**G**) SH-SY5Y cells were exposed to rotenone with or without apelin-13, or with APJ siRNA pretreatment prior to rotenone and apelin-13. Western blot was applied to detect the expression of LC3B-II/LC3B-I, p62, and α-synuclein. Apelin-13 significantly increased the ratio of LC3B-II/LC3B-I, and reduced the expression of p62 and α-synuclein in rotenone-treated SH-SY5Y cells; APJ siRNA pretreatment abolished these effects. Data are presented as mean ± SEM of three replications. * *p* < 0.05; ** p < 0.01 compared with the control; ^##^ *p* < 0.01 compared with the rotenone group; ^△^ *p* < 0.05; ^△△^ *p* < 0.01 compared with the Apelin-13+Rotenone group.

Consistent with data in Figure 3A–C, apelin-13 increased the ratio of LC3B-II/LC3B-I, and decreased p62 levels in SH-SY5Y cells. APJ siRNA was able to abolish these effects of apelin-13 (Figure 11A–C). APJ siRNA3 was used in the following experiments. Similarly, apelin-13 induced autophagy in rotenone treated cells, as indicated by higher ratio of LC3B-II/LC3B-I, and that less p62 was blocked with APJ siRNA pretreatment (Figure 11D–G). These data suggested that apelin-13 activated autophagy via the receptor APJ in this model.

**Figure 12:**
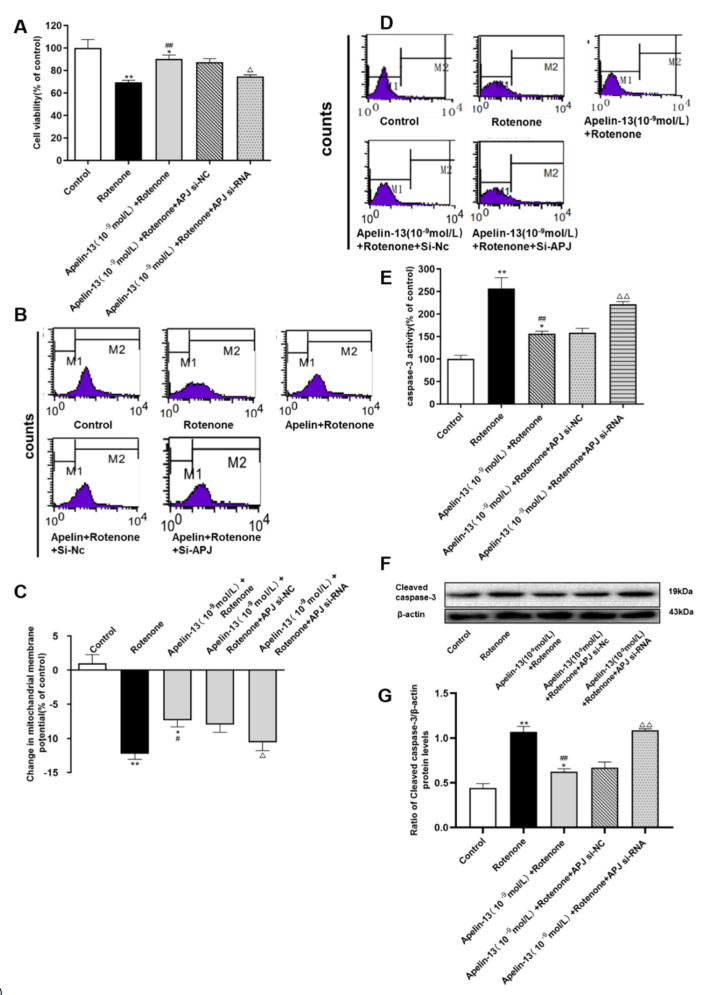
Effects of APJ siRNA on the neuroprotective effects of apelin-13 in SH-SY5Y cells. (**A**) The cell viability was tested by MTT assays, and is presented as percentage of control. Apelin-13 rescued rotenone-induced cell death, and this effect was prevented by APJ siRNA. (**B**,**C**) Fluorometric assays of ΔΨm and statistical analysis. Apelin-13 antagonized rotenone-induced reduction in △ΨM, and pre-incubation with APJ siRNA abolished the reduction. (**D**,**E**) Fluorometric assay of caspase-3 activity and statistical analysis. Apelin-13 could antagonize rotenone-induced caspase-3 activation, and pre-incubation with APJ siRNA abolished the activation. (**F**,**G**) Western blot was applied to detect the expression of cleaved caspase-3. Apelin-13 antagonize the increase induced by rotenone in the expression of cleaved caspase-3 protein; APJ siRNA pretreatment abolished these changes. Data are presented as mean ± SEM of three replications. * *p* < 0.05; ** p < 0.01 compared with the control; ^#^ *p* < 0.05; ^##^ *p* < 0.01 compared with the rotenone group; ^△^ *p* < 0.05; ^△△^ *p* < 0.01 compared with the Apelin-13+Rotenone group.

We then further explored whether apelin-13 exerted its protective effect via the receptor APJ in SH-SY5Y cells. As shown in Figure 12A–C, the cell viability and ΔΨm were rescued by apelin-13 in the rotenone-treated SH-SY5Y cells, in which APJ siRNA could abolish these effects. Apelin-13 could antagonize rotenone-induced caspase-3 activation and cleaved caspase-3 protein expression, and pre-incubation with APJ siRNA abolished these protective effects (Figure 12D–G).

### 2.8. Apelin-13 Antagonized Rotenone Induced Dopaminergic Neurons Loss and Restored the Contents of Dopamine and Its Metabolites in the Str in Rats

**Figure 13:**
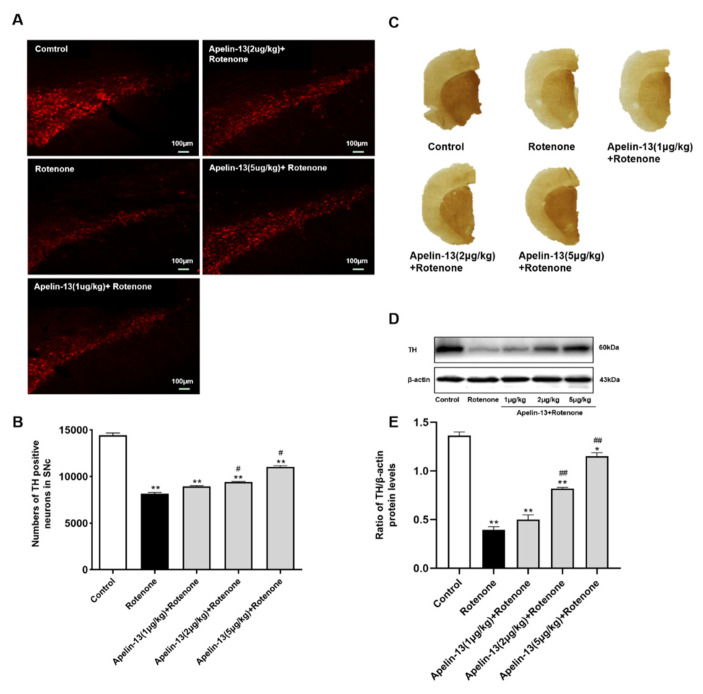
Effects of apelin-13 on the degeneration of nigrostriatal dopaminergic neurons in rats with rotenone administration. The in vivo Parkinson’s disease (PD) model was established in Wistar rats by the administration of rotenone. (**A**,**B**) The TH-positive cells in the substantia nigra (SN) of the rats was analyzed by immunofluorescence analysis. The number of TH immunopositive cells decreased in the SN of rats in the rotenone group; this effect was attenuated by apelin-13 (2 μg/kg and 5 μg/kg) administration. Data are presented as mean ± SEM of ten replications. * *p* < 0.05; ** *p* < 0.01 compared with the control; ^#^ *p* < 0.05; ^##^*p* < 0.01 compared with the rotenone group. Bar = 100 μm. (**C**) The expression of TH in the striatum (Str) of the rats was assessed by immunohistochemistry analysis. Weaker staining of TH was observed in rats with rotenone administration when compared to the control; however, a more robust staining was observed in the apelin (5 μg/kg) + rotenone group. (**D**,**E**) The expression of TH in the SN was measured by Western blot analysis. TH protein levels were down-regulated in the rotenone group; these effects were attenuated by apelin-13 (2 μg/kg and 5 μg/kg) administration. Data are presented as mean ± SEM of three replications. * *p* < 0.05; ** *p* < 0.01 compared with the control; ^#^ *p* < 0.05; ^##^ *p* < 0.01 compared with the rotenone group.

**Figure 14:**
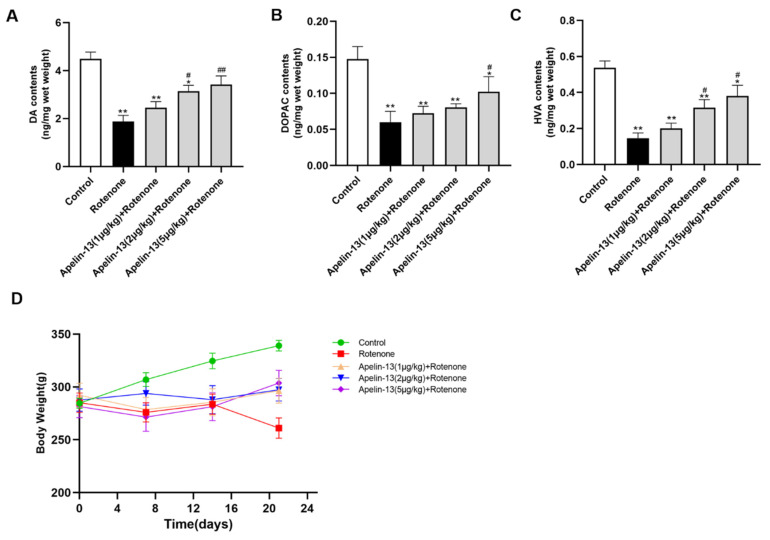
Effects of apelin-13 on the content of DA and its metabolites in the Str of rats with rotenone administration. (**A**–**C**) The levels of DA, DOPAC, and HVA were analyzed by HPLC analysis in the str. There was a reduction of DA, DOPAC, and HVA levels in the rotenone group. DA, DOPAC, and HVA levels were restored in the Apelin-13 (5 μg/kg) + rotenone and Apelin-13 (2 μg/kg) + rotenone groups; DA and HVA levels were restored in the Apelin-13 (2 μg/kg) + rotenone group. (**D**) The body weight of the rats was recorded, and is shown. Rotenone induced a loss of body weight, which was fully abolished in the Apelin-13 (1 μg/kg, 2 μg/kg, and 5 μg/kg) + rotenone groups. Data are presented as mean ± SEM of twelve replications. * *p* < 0.05; ** *p* < 0.01 compared with the control; ^#^ *p* < 0.05; ^##^ *p* < 0.01 compared with the rotenone group.

We established an in vivo PD rat model by the administration of rotenone (3 mg/kg). Immunofluorescence analysis showed that the tyrosine hydroxylase (TH)-positive cells in the SN of the rats were reduced by rotenone administration, while apelin-13 (2 μg/kg and 5 μg/kg) was able to rescue the loss of TH-positive cells in the SN, whereas 1 μg/kg apelin-13 did not show any effects (Figure 13A,B). Accordingly, immunohistochemistry analysis revealed that apelin-13 (2 μg/kg and 5 μg/kg) restored TH staining in the Str (Figure 13C), as well as TH protein levels in the SN (Figure 13D,E). We then assessed the impact of apelin-13 on the contents of dopamine and its metabolites. The results showed that the administration of rotenone reduced the levels of dopamine (DA) and its metabolites DOPAC and HVA in the Str. Apelin-13 (5 μg/kg) could restore the levels of DA, DOPAC, and HVA; apelin-13 (2 μg/kg) could restore the levels of DA and HVA, while apelin-13 (1 μg /kg) did not show any effects (Figure 14A–C). We also observed that apelin-13 administration was able to restore the bodyweight reduction induced by the rotenone in the rats (Figure 14D). Taken together, these data suggest that apelin-13 can alleviate nigrostriatal dopaminergic neuron degeneration in vivo.

## 3. Discussion

In our study, we demonstrated that apelin-13 protects SH-SY5Y dopaminergic cells against rotenone-induced cell injury and apoptosis. Apelin-13 could induce autophagy in both normal and rotenone-treated SH-SY5Y cells, which might be mediated by the stimulation of AMPK/mTOR/ULK1 signaling via its receptor-APJ. The protective effects of apelin-13 against rotenone induced neurotoxicity was also observed in rats.

Tatemoto et al. discovered the satiety neuropeptide, apelin, in 1998 [31], which is an orphan G protein-coupled apelin receptor (APJ) ligand [12]. This novel brain–gut peptide can regulate food intake and energy homeostasis in the hypothalamus [32]. Studies have shown that apelin-13 is able to penetrate the blood–brain barrier via an unsaturated mechanism, which indicates that it is feasible to transport to the central nervous system pharmacologically through peripheral pathways [33]. Apelin-13 is a neuropeptide which is capable of protecting against neuronal apoptosis and excitotoxicity in vitro, and is associated with neuronal survival in vivo following cerebral ischemia or ischemia/reperfusion injury, amyotrophic lateral sclerosis (ALS), and seizure [34,35,36]. In addition, apelin-13 has an attenuating effect on the expression of pro-inflammatory cytokines, and inhibits microglia, astrocytes, and other inflammatory cells [37,38]. As a neuropeptide, several roles of apelin-13 in the modulation of PD development have been reported. It has been identified that apelin-13 inhibits motion impairment and limits the variations in synaptic plasticity-associated particles in the Str of PD rats [39]. Apelin-13 attenuated cognitive impairment in 6-hydroxydopamine-produced SN lesions in the rats, as well as antagonized 6-hydroxydopamine-produced neurotoxicity in SH-SY5Y cells [16,40]. Apelin-13 also showed anti-apoptotic properties against neurotoxins MPP+ and methamphetamine^41^. Mitochondrial dysfunction played an important role in the etiology of PD [41,42]. Rotenone is an environmental neurotoxin that impairs ATP synthesis, and results in mitochondrial dysfunction [43,44,45], which induced the release of cytochrome c, and then promoted cell damage through stimulating a caspase-dependent apoptosis signal. In our study, rotenone induced dopaminergic neuronal death in rats, decreased the cell viability, damaged mitochondrial function, and then promoted apoptosis, in SH-SY5Y cells. Apelin-13 blocked rotenone-induced cell injury, prevented ΔΨm and Bcl-2/Bax reduction, and antagonized caspase-3 activation and morphological changes in the nuclei of rotenone-treated SH-SY5Y cells. These results indicated that apelin-13 antagonized rotenone-induced dopaminergic neuron apoptosis by ameliorating mitochondrial dysfunction. We also demonstrated that Intracerebroventricular injection (ICV) of apelin-13 (1 μg/kg, 2 μg/kg, 5 μg/kg) once a day for 26 consecutive days effectively rescued dopaminergic neuronal loss, and prevented DA depletion in the striatum of rats administrated with rotenone.

The mechanisms underlying the neuroprotective effects of apelin-13 have not been fully elucidated. Increasing evidence indicates that autophagy impairment plays a crucial role in PD progression [46]. The autophagy system can effectively remove some damaged organelles, such as damaged mitochondria and misfolded proteins; while dysfunction of autophagy may induce protein accumulation and cell death [47]. On the contrary, the inhibition of autophagy increases the transfer and release of SNCA/α-synuclein by extracellular blisters, thus autophagy activation is critical to the attenuation of PD progression [48]. Bioactive compounds that potentially modulate autophagy are increasingly suggested as new drugs for therapeutic purposes in PD [49]. For instance, the induction of autophagy by rapamycin, a well-known autophagy inducer, contributed to a prominent protection against rotenone-induced toxicity, and prevented the formation of poly-ubiquitinated protein aggregates [50]. Several natural compounds, such as kaempferol, celastrol, and resveratrol, could enhance autophagy, and thus exert neuroprotective effects in PD models [51,52,53,54,55]. More recently, long noncoding RNA HAGLROS modulated autophagy and apoptosis by controlling PI3K/Akt/mTOR activation and miR-100/ATG10 signaling in PD [56]. In the brain, apelin-13 protected dopaminergic neurons in MPTP-induced PD model mice in vivo, through inhibiting ERS and promoting autophagy [17]. However, there has been a controversial report that apelin-13 relieves traumatic brain damage-produced injury through repressing autophagy [57]. We hypothesized that the neuroprotective property of apelin-13 might be associated with the regulation of autophagy. As we expected, apelin-13 increased the ratio of LC3B-II/LC3B-I, and promoted the formation of massive LC3B-II puncta, meanwhile, it reduced p62 levels in SH-SY5Y cells. With CQ pre-treatments with aplin-13, SH-SY5Y cells showed a further increase in the ratio of LC3B-II/LC3B-I, and higher levels of p62, suggesting that the enhancement of autophagy markers by apelin-13 is due to induction of autophagy rather than blockage of autophagosome maturation. More importantly, apelin-13 induced autophagy in rotenone treated cells, as indicated by the higher ratio of LC3B-II/LC3B-I, less p62, and α-synuclein accumulated in the cells. Autophagy dysfunction was supposed in α-synuclein aggregation, therefore, clearance of aggregated α-synuclein, via up-regulation of the autophagy-lysosomal pathway, could provide a pharmacologically viable approach to the treatment of PD [58,59]. Considering the presence of autophagy impairment in rotenone models, these results indicate that apelin-13 might serve as an autophagy inducer and exert protective effects against rotenone-induced neurotoxicity. This was confirmed by the result that autophagy inhibitor 3-MA abolished the restoration of mitochondrial function, and the anti-apoptotic effects exerted by apelin-13.

One of the major transduction pathways for apelin-13 signals depends on the interaction with a Gi-protein coupled to the APJ receptor and protein AMPK [60,61]. AMPK/mTOR/ULK1 signaling has been identified in the modulation of autophagy. It has been well recognized that the activation of AMPK/mTOR/ULK1 signaling is required for autophagy induction [62]. AMPK is a major energy-sensing kinase, which activates many catabolic processes in multicellular organisms. AMPKα could also be involved in regulating autophagy. It was proven to have an essential role for regulating autophagic signals in human hepatocytes, HT-29 cells, and HeLa cells [63]. A recent study indicated that the yeast ortholog of AMPKα (SNF1) can activate the autophagy system [64]. Activation of AMPKα happens, not only by low energy levels in cells, but also by all kinds of other non-starvation-related autophagy inducers, even under normal energy levels [65]. Both mTORC1 and AMPKα are able to regulate autophagy inducing complex Ulk1/2 kinase activity by direct phosphorylation. On the one hand, activation of AMPK, to oppositely regulate mTORC1, and mTORC1 inactivation in turn, led to Ulk1/2 kinase activity [62]. On the other hand, it was in a direct interaction between AMPKα and Ulk1 that AMPKα activation positively up-regulated Ulk1 activity [66,67]. We then explored whether apelin-13 induces autophagy by stimulating AMPK/mTOR/ULK1 signaling in SH-SY5Y cells. Apelin-13 could stimulate AMPK activity in myocardial microvascular endothelial cells [68]; it up-regulated AMPK phosphorylation levels in cerebral ischemia insults, and AMPK signals were involved in the mechanism of apelin-13-mediated neuroprotection [69]. Consistent with previous studies, our data showed that apelin-13 treatment of SH-SY5Y cells up-regulated AMPKα and ULK1 phosphorylation levels, while the phosphorylation of mTOR protein was inhibited. AMPKα inhibitor compound C efficiently blocked the effects of apelin-13 on the AMPK/mTOR/ULK1 signaling pathway. Compound C also blocked apelin-13-induced autophagy activation in rotenone-treated cells [70], as indicated by its suppression on the increased ratio of LC3B-II/LC3B-I, inhibited p62, and the α-synuclein levels induced by apelin-13. Accordingly, pre-incubation with compound C abolished the protective effects induced by apelin-13. Taken together, these data suggest that apelin-13 exerts its protective effect on the SH-SY5Y cells from rotenone-induced injury and apoptosis by the stimulation of AMPK/mTOR/ULK1 signaling-mediated autophagy.

First, a type of receptor protein, APJ, related to the angiotensin type 1 receptor (AT1), and a new G protein-coupled receptor (GPCR) family, termed “orphan receptors” [71], were discovered. Then apelin was believed to be the endogenous ligand of APJ, based on the fact that it was extracted from the secretions of cattle stomach tissues and named apelin (APJ endogenous ligand) [31]. The apelin/APJ system is confirmed to be a signal for arteriogenesis in hepatocellular carcinoma [72]. The APJ receptor antagonist F13A was used to block apelin/APJ signaling to inhibit tumor growth and small arteries in an HCC subcutaneous murine tumor model [73]. Similarly, treatment of LoVo cells, (a human colon adenocarcinoma cell line) with APJ receptor antagonist significantly reduced the cell proliferation rate [73]. Apelin-13 and its binding receptor, APJ, are widely expressed in CNS, especially in the hypothalamus, hippocamp, cortex, and substantia nigra [71,74,75,76]. In the present study, the efficiency of APJ depletion by APJ siRNA was validated in the cells. The results show that APJ siRNA were able to block the effects of apelin-13 on AMPK/mTOR/ULK1 signaling. Meanwhile, APJ siRNA was able to inhibit apelin-13 activated autophagy and the protective effects.

In conclusion, we revealed that apelin-13 could activate AMPK/mTOR/ULK1 signaling in the rotenone-treated SH-SY5Y cells, and induce autophagy and protect SH-SY5Y cells from rotenone-induced injury. As a kind of brain–gut peptide, apelin-13 was able to active autophagy, even in the presence of the neurotoxin, rotenone. Therefore, these results reinforce the possibility of activating autophagy as a therapeutic target, and explore the promising strategies related to autophagy activation.

## 4. Materials and Methods

### 4.1. Materials

Unless otherwise stated, all chemicals including rotenone, 3-Methyladenine (3-MA), and chloroquine (CQ) were purchased from Sigma Chemical Co (St Louis, MO, USA). Compound C was purchased from Selleck (TX, USA). Apelin-13 was purchased from Santa Cruz Biotechnology, Inc. (Dallas, TX, USA). The primary LC3B antibody was purchased from Novus Biologicals (Littleton, CO, USA). The primary tyrosine hydroxylase (TH) antibody was purchased from Millipore (Darmstadt, Germany). The primary α-synuclein, p62, AMPK, p-AMPK, ULK-1, and p-ULK-1 antibody for Western blot, were from Cell Signaling Technology (Boston, CM, USA). Beta-actin antibody was from Bioss (Beijing, China). The goat anti-rabbit IgG-horseradish peroxidase secondary antibodies from Absin (Shanghai, China). The goat anti-rabbit IgG (Alexa Fluor-488, green) secondary antibody was from Invitrogen (Eugene, OR, USA). Dulbecco’s modified Eagle’s medium Nutrient Mixture-F12 (DMEM/F12) was from Gibco (Gibco, Grand Island, NY, USA). The PE-conjugated monoclonal active caspase-3 antibody apoptosis kit was from BD Bioscience Company (San Diego, CA, USA). Hoechst 33258 and the BCA kit were from Beyotime (Jiangsu, China). Other chemicals and regents were available from local commercial sources.

### 4.2. Cell Culture and Treatment

The human neuroblastoma SH-SY5Y cells, purchased from the Cell Bank of Chinese Academy of Sciences (Shanghai, China), were grown in DMEM/F12 (Gibco, Grand Island, NY, USA), containing growth medium supplemented with 10% FBS, 100 U/mL penicillin, and 100 lg/mL streptomycin in a humidified atmosphere containing 5% CO_2_ at 37 °C. The cells were cultured in the medium of DMEM/F12 containing 10% fetal bovine serum (Gibco, Grand Island, NY, USA), 0.1 mg/mL streptomycin (Solarbio, Beijing, China), and 100 units/mL penicillin (Solarbio, Beijing, China) at a condition of 37 °C with 5% CO_2_. An in vitro PD model was established in the SH-SY5Y cells by the treatment of rotenone (Sigma, St. Louis, MO, USA) at the indicated dose for 24 h. The cells were treated with apelin-13, 3-MA, compound C, apelin receptor (APJ) siRNA (GenePharma, Shanghai, China), or CQ (a lysosome inhibitor), as described in the results. The transfection in the cells was performed by Liposome 3000 (Invitrogen, Carlsbad, CA, USA), according to the manufacturer’s instructions.

### 4.3. MTT Assays

MTT assays measured the cell viability of SH-SY5Y cells. Briefly, about 2×10^4^ SH-SY5Y cells were put into 96 wells and cultured for 24 h. After treatment, the cells were added to with a 20 μL MTT solution (5 mg/mL) (Sigma, St. Louis, MO, USA), and cultured for an extra 4 h discarded medium, and 150 μL/well DMSO (Thermo, Waltham, MA, USA) was used to treat the wells. The optical densities of the standards and samples were measured by subtracting the readings at 540 nm from the readings at 450 nm using a multifunctional microplate reader (SpectraMax M5, Molecular Devices, San Jose, CA, USA).

### 4.4. Assessment of ΔΨm and Caspase-3 Activation

To evaluate the ΔΨm during the rotenone-induced mitochondria dysfunction, SH-SY5Y cells were treated with HBS and rhodamine123 (Sigma, St. Louis, MO, USA, 5 μmol/L). The changes of the mitochondrial membrane potential with various treatments were monitored by flow cytometry, as reported previously. Rhodamine-123 as a cationic fluorescent probe can penetrate into mitochondria, which is an indicator of the MMP. After pretreatment with apelin (1 × 10^−9^ mol/L) for 30 min, cells were subjected to 500 nmol/L rotenone in serum-contained DMEM for 24 h, and then incubated with rhodamine123 in a final concentration of 5 mol/L for 30 min at 37 °C. Then untreated controls and treated cells were harvested. After washing twice with HBS, the cells were resuspended in 1 mL HBS. For analysis, fluorescence intensity was recorded at 488-nm excitation and 525-nm emission wavelengths. Results are shown as FL1-H (Fluorescence 1-histogram), setting the gated region M1 and M2 as a marker to assess changes of fluorescence intensity, using Cell Quest software (BD Bioscience). SH-SY5Y Cells were seeded at 1 × 10^5^ cells/mL into 6-well plates and treated as described above. Caspase-3 activity was assessed using a PE-conjugated monoclonal active caspase-3 antibody apoptosis kit, according to the manufacturer’s protocol for flow cytometry. After washing twice with cold phosphate-buffered saline (PBS), the cells were then resuspended in Citofix/CytopermTM solution at a concentration of 1 × 10^6^ cells/0.5 mL. The samples were incubated for 30 min at 37 °C, and washed twice with perm/wash buffer. Cells were resuspended with 0.5 mL perm/wash buffer and thereafter analyzed using a BD FACS caliber flow cytometer (BD Biosciences, San Diego, CA, USA). The percentage of positive cells reacting with the antibody was examined with Cellguest Software (BD Bioscience, San Diego, CA, USA).

### 4.5. Hoechst 33258 Staining

To assess the neuroprotective effects of apelin-13 against rotenone induced apoptosis, nuclear morphology was detected using a previously described method in our lab [77,78]. SH-SY5Y cells were cultured on coverslips in 24-well plates. Cells were fixed in 4% paraformaldehyde (PFA) for 30 min, washed twice with PBS, and stained with Hoechst 33258 dye for 15 min at room temperature. After washing 3 times to remove the excess dye, cells were examined and photographed under a confocal laser scanning microscope (Fluoview FV500, Olympus, Osaka, Japan). Based on changes in nuclear morphology, such as chromatin condensation and fragmentation, apoptotic cells are defined. We delineated a 400 μm^2^ frame in each space, and then selected 10 different regions to calculate all condensed nuclei and normal nuclei. Then we calculated the average sum of the condensed nuclei and normal nuclei for each hole. The data were presented as percentages, and the proportion of condensed nuclei relative to the total number of nuclei was calculated.

### 4.6. Western Blot Analysis

Samples from cells and animals were prepared in ice-cold radio immunoprecipitation assay (RIPA) lysis buffer with a protease inhibitor (CWBIO, Beijing, China). The protein concentration of the samples was then determined using a BCA assay (Thermo, USA). Protein samples (20 μg) were loaded and separated by 8% or 10% sodium dodecyl sulfate polyacrylamide gel electrophoresis (SDS-PAGE) at 80 V for 30 min, followed by 120 V for 90 min. The proteins were subsequently transferred to PVDF membranes (Millipore Corp, Billerica, MA, USA) by electroblotting. After 2 h blocking with 10% non-fat milk in PBST (80 mm Na2HPO4, 20 mm NaH2PO4, 100 mm NaCl, 0.1% Tween 20, pH 7.4) at room temperature, the membranes were incubated overnight at 4 °C with TH (1:4000), α-synuclein (1:1000), Bax (1:1000), Bcl-2 (1:1000), caspase-3 (1:1000), LC3B (1:2000), p62 (1:1000), AMPKα (1:1000), ULK1 (1:1000), mTOR (1:1000), p-AMPKα (1:1000), p-ULK1 (1:1000), p-mTOR (1:1000), and β-actin (1:5000), in which β-actin served as the control. The membranes were washed three times for 10 min each using PBST, incubated with the appropriate goat anti-rabbit IgG-horseradish peroxidase secondary antibodies (1:5000) at room temperature for 1 h, and washed three more times in PBST buffer. The blots were stained with the Clarity Western enhanced chemiluminescence (ECL) substrate (Millipore Corp, Billerica, MA, USA), and target bands were visualized using a UVP BioDoc-It Imaging System (Upland, CA, USA). The target bands were quantified using ImageJ software (NIH Image, Bethesda, MD, USA), and the density of each band was normalized against Beta-Actin.

### 4.7. Animals and Treatment

All procedures were carried out in accordance with the National Institute of Health Guide for the Care and Use of Laboratory Animals, and were approved by the Animal Ethics Committee of Qingdao University (20181027, 27 October 2018). Briefly, adult Wistar rats (male) weighing 250 ~ 300 g were reared at room temperature for 12 h, and with free access to food and water. Rats were randomly separated into five groups: (1) control group: rats received intracerebroventricular injection (ICV) saline injection only; (2) rotenone-treated group: rats received rotenone (3 mg/kg, intraperitoneal injection, i.p.) and ICV saline injections once per day for 21 consecutive days; (3) apelin-13-pretreated group: rats were pretreated with different doses of apelin-13 (1 μg/kg, 2 μg/kg, and 5 μg/kg, ICV) once per day for 26 consecutive days, and received rotenone (3 mg/kg, i.p.) for the final 21 days. Rotenone was freshly prepared using DMSO as a solvent and medium-chain triglyceride (MCT) at a 1:49 ratio with a final concentration of 3 mg/kg.

### 4.8. Immunofluorescence and Immunohistochemistry Staining

SH-SY5Y cells were grown on sterile Poly-D-Lysine-coated slides in 12-well plates and pre-treated with 10^−9^ mol/L apelin-13 for 30 min. Then cells were treated with vehicle or 500 nM rotenone for 24 h respectively, followed by washing once with 0.01% PBS (pH 7.4). The coverslips were fixed in 4% PFA for 10 min with 0.1% Triton X-100. Cells were rinsed three times (5 min each) in PBS and blocked with 5% goat serum in PBS for 1 h at room temperature. After blocking, they were incubated overnight at 4 °C with the primary antibodies against LC3B (1:300). The samples were then washed three times (5 min each) with PBS and incubated with goat anti-rabbit IgG (Alexa Fluor-488, green) secondary antibody (1:500) for 1 h at RT in the dark, followed by staining the nuclei with Hoechst 33258 for 15 min. Finally, LC3 expression in cells was detected under a fluorescence microscope (Observer A1, Zeiss, Germany). The brain was fixed in 4% PFA at 4 °C for 72 h, and then incubated in 0.1 M phosphate buffer (pH 7.4) containing 25% sucrose at 4 °C for 2 to 3 days. Then the frozen brain was cut into 25-μm-thick sections and brain tissues sections were used for immunofluorescence staining of SN and immunohistochemical staining of Str. First, the sections were incubated with 0.1% Triton X-100 in phosphate buffered saline (PBS) for 24 h. Second, the sections were incubated overnight at 4 °C with TH primary antibody (Millipore Corporation, USA, 1:2000) in PBS containing 0.1% Triton X-100. Third, the sections for SN staining were incubated with Alexa Fluor 555 donkey anti-rabbit secondary antibody, and images were obtained with an immunofluorescence microscope (Observer A1, Zeiss, Germany) at 200 and 400× magnifications. Then the sections for Str staining were incubated with biotinylated goat anti-rabbit IgG at 37 °C for 1 h. Finally, diaminobenzidine hydrogen peroxide (0.01%) was used as a coloring agent, and a camera was used to obtain a digital image.

### 4.9. Measurement of Dopamine and Its Metabolites Levels by HPLC

The bodyweight of the rats was recorded and calculated. Twelve rats from each group provided samples for HPLC. Samples were weighed and then homogenized in 0.3 mL liquid A (0.4 M perchloric acid). After initial centrifugation (120,000 rpm for 20 min at 4 °C), 80 μL of the supernatant was transferred into Eppendorf tubes, and 40 μL liquid B (20 mM citromalic acid potassium, 300 mM dipotassium phosphate, 2 mM EDTA-2Na) was added. After additional centrifugation (120,000 rpm for 20 min at 4 °C), 100 μL of the supernatant was assayed for DA and its metabolites, DOPAC and HVA, by HPLC. Separation was achieved on a PE C18 reverse-phase column. The mobile phase consisted of 20 mM citromalic acid, 50 mM sodium caproate, 0.134 mM EDTA-2Na, 3.75 mM sodium octane sulphonic acid, and 1 mM di-sec-butylamine at 5% (*v*/*v*) methanol; the flow-rate was 1 mL/min. A 2465 electrochemical detector (Waters, Milford, MA, USA) was operated in screen mode. The results were expressed as ng/mg wet weight of brain tissue.

### 4.10. Statistical Analysis

Data were presented as mean ± SEM, and the statistical analysis was performed by SPSS software (version 18.0). The one-way ANOVA was applied for comparing among multiple groups. *p* < 0.05 were considered as statistically significant.

## Abbreviations

PD	Parkinson’s disease
TH	Tyrosine hydroxylase
SN	Substantia nigra
Str	Striatum
ΔΨm	Mitochondrial membrane potential
LC3B	Light chain 3B
APJ	Apelin receptor
ROS	Reactive oxygen species
3-MA	3-Methyladenine
ICV	Intracerebroventricular injection

## References

[1] Rocha E.M., De Miranda B., Sanders L.H. (2018). Alpha-synuclein: Pathology, mitochondrial dysfunction and neuroinflammation in Parkinson’s disease. Neurobiol. Dis..

[2] Hewitt V.L., Whitworth A.J. (2017). Mechanisms of Parkinson’s Disease: Lessons from Drosophila. Curr. Top. Dev. Biol..

[3] Kaur R., Mehan S., Singh S. (2019). Understanding multifactorial architecture of Parkinson’s disease: Pathophysiology to management. Neurol. Sci. Off. J. Ital. Neurol. Soc. Ital. Soc. Clin. Neurophysiol..

[4] Lipski J., Nistico R., Berretta N., Guatteo E., Bernardi G., Mercuri N.B. (2011). L-DOPA: A scapegoat for accelerated neurodegeneration in Parkinson’s disease?. Prog. Neurobiol..

[5] Calsolaro V., Edison P. (2015). Novel GLP-1 (Glucagon-Like Peptide-1) Analogues and Insulin in the Treatment for Alzheimer’s Disease and Other Neurodegenerative Diseases. CNS Drugs.

[6] St-Gelais F., Jomphe C., Trudeau L.E. (2006). The role of neurotensin in central nervous system pathophysiology: What is the evidence?. J. Psychiatry Neurosci..

[7] Duarte-Neves J., Pereira de Almeida L., Cavadas C. (2016). Neuropeptide Y (NPY) as a therapeutic target for neurodegenerative diseases. Neurobiol. Dis..

[8] Shen X.L., Song N., Du X.X., Li Y., Xie J.X., Jiang H. (2017). Nesfatin-1 protects dopaminergic neurons against MPP(+)/MPTP-induced neurotoxicity through the C-Raf-ERK1/2-dependent anti-apoptotic pathway. Sci. Rep..

[9] Jiang H., Li L.J., Wang J., Xie J.X. (2008). Ghrelin antagonizes MPTP-induced neurotoxicity to the dopaminergic neurons in mouse substantia nigra. Exp. Neurol..

[10] Shi L., Bian X., Qu Z., Ma Z., Zhou Y., Wang K., Jiang H., Xie J. (2013). Peptide hormone ghrelin enhances neuronal excitability by inhibition of Kv7/KCNQ channels. Nat. Commun..

[11] Liu L., Xu H., Jiang H., Wang J., Song N., Xie J. (2010). Ghrelin prevents 1-methyl-4-phenylpyridinium ion-induced cytotoxicity through antioxidation and NF-kappaB modulation in MES23.5 cells. Exp. Neurol..

[12] Antushevich H., Wojcik M. (2018). Review: Apelin in disease. Clin. Chim. Acta.

[13] Kumar P., Ashokan A., Aradhyam G.K. (2016). Apelin binding to human APJ receptor leads to biased signaling. Biochim. Biophys. Acta.

[14] Mughal A., O’Rourke S.T. (2018). Vascular effects of apelin: Mechanisms and therapeutic potential. Pharmacy.

[15] Kurowska P., Barbe A., Rozycka M., Chmielinska J., Dupont J., Rak A. (2018). Apelin in Reproductive Physiology and Pathology of Different Species: A Critical Review. Int. J. Endocrinol..

[16] Pouresmaeili-Babaki E., Esmaeili-Mahani S., Abbasnejad M., Ravan H. (2018). Protective Effect of Neuropeptide Apelin-13 on 6-Hydroxydopamine-Induced Neurotoxicity in SH-SY5Y Dopaminergic Cells: Involvement of Its Antioxidant and Antiapoptotic Properties. Rejuvenation Res..

[17] Zhu J., Dou S., Jiang Y., Chen J., Wang C., Cheng B. (2019). Apelin-13 protects dopaminergic neurons in MPTP-induced Parkinson’s disease model mice through inhibiting endoplasmic reticulum stress and promoting autophagy. Brain Res..

[18] Meredith G.E., Sonsalla P.K., Chesselet M.F. (2008). Animal models of Parkinson’s disease progression. Acta Neuropathol..

[19] Menzies F.M., Fleming A., Caricasole A., Bento C.F., Andrews S.P., Ashkenazi A., Füllgrabe J., Jackson A., Jimenez Sanchez M., Karabiyik C. (2017). Autophagy and Neurodegeneration: Pathogenic Mechanisms and Therapeutic Opportunities. Neuron.

[20] Cerri S., Blandini F. (2019). Role of Autophagy in Parkinson’s Disease. Curr. Med. Chem..

[21] Bellomo G., Paciotti S., Gatticchi L., Parnetti L. (2020). The vicious cycle between α-synuclein aggregation and autophagic-lysosomal dysfunction. Mov. Disord. Off. J. Mov. Disord. Soc..

[22] Lin K.J., Lin K.L., Chen S.D., Liou C.W., Chuang Y.C., Lin H.Y., Lin T.K. (2019). The Overcrowded Crossroads: Mitochondria, Alpha-Synuclein, and the Endo-Lysosomal System Interaction in Parkinson’s Disease. Int. J. Mol. Sci..

[23] Pan T., Kondo S., Zhu W., Xie W., Jankovic J., Le W. (2008). Neuroprotection of rapamycin in lactacystin-induced neurodegeneration via autophagy enhancement. Neurobiol. Dis..

[24] Xiong N., Jia M., Chen C., Xiong J., Zhang Z., Huang J., Hou L., Yang H., Cao X., Liang Z. (2011). Potential autophagy enhancers attenuate rotenone-induced toxicity in SH-SY5Y. Neuroscience.

[25] Jiao H., Zhang Z., Ma Q., Fu W., Liu Z. (2013). Mechanism underlying the inhibitory effect of Apelin-13 on glucose deprivation-induced autophagy in rat cardiomyocytes. Exp. Ther. Med..

[26] Yang L., Su T., Lv D., Xie F., Liu W., Cao J., Sheikh I.A., Qin X., Li L., Chen L. (2014). ERK1/2 mediates lung adenocarcinoma cell proliferation and autophagy induced by apelin-13. Acta Biochim. Et Biophys. Sin..

[27] Yao F., Lv Y.C., Zhang M., Xie W., Tan Y.L., Gong D., Cheng H.P., Liu D., Li L., Liu X.Y. (2015). Apelin-13 impedes foam cell formation by activating Class III PI3K/Beclin-1-mediated autophagic pathway. Biochem. Biophys. Res. Commun..

[28] Yang Y., Zhang X.J., Li L.T., Cui H.Y., Zhang C., Zhu C.H., Miao J.Y. (2016). Apelin-13 protects against apoptosis by activating AMP-activated protein kinase pathway in ischemia stroke. Peptides.

[29] Curry D.W., Stutz B., Andrews Z.B., Elsworth J.D. (2018). Targeting AMPK Signaling as a Neuroprotective Strategy in Parkinson’s Disease. J. Parkinsons Dis..

[30] Chen S., Guo D., Lei B., Bi J., Yang H. (2020). Biglycan protects human neuroblastoma cells from nitric oxide-induced death by inhibiting AMPK-mTOR mediated autophagy and intracellular ROS level. Biotechnol. Lett..

[31] Tatemoto K., Hosoya M., Habata Y., Fujii R., Kakegawa T., Zou M.X., Kawamata Y., Fukusumi S., Hinuma S., Kitada C. (1998). Isolation and characterization of a novel endogenous peptide ligand for the human APJ receptor. Biochem. Biophys. Res. Commun..

[32] Mashaqi S., Badr M.S. (2019). The Impact of Obstructive Sleep Apnea and Positive Airway Pressure Therapy on Metabolic Peptides Regulating Appetite, Food Intake, Energy Homeostasis, and Systemic Inflammation: A Literature Review. J. Clin. Sleep Med. JCSM Off. Publ. Am. Acad. Sleep Med..

[33] Lee T.H., Cheng K.K., Hoo R.L., Siu P.M., Yau S.Y. (2019). The Novel Perspectives of Adipokines on Brain Health. Int. J. Mol. Sci..

[34] Kasai A., Kinjo T., Ishihara R., Sakai I., Ishimaru Y., Yoshioka Y., Yamamuro A., Ishige K., Ito Y., Maeda S. (2011). Apelin deficiency accelerates the progression of amyotrophic lateral sclerosis. PLoS ONE.

[35] Sabry M.M., Ramadan N.M., Al Dreny B.A., Rashed L.A., Abo El Enein A. (2019). Protective effect of apelin preconditioning in a rat model of hepatic ischemia reperfusion injury; possible interaction between the apelin/APJ system, Ang II/AT1R system and eNOS. United Eur. Gastroenterol. J..

[36] Kalantaripour T.P., Esmaeili-Mahani S., Sheibani V., Asadi-Shekaari M., Pasban-Aliabadi H. (2016). Anticonvulsant and neuroprotective effects of apelin-13 on pentylenetetrazole-induced seizures in male rats. Biomed. Pharmacother. Biomed. Pharmacother..

[37] Zhou S., Chen S., Xie W., Guo X., Zhao J. (2020). Microglia polarization of hippocampus is involved in the mechanism of Apelin-13 ameliorating chronic water immersion restraint stress-induced depression-like behavior in rats. Neuropeptides.

[38] Xin Q., Cheng B., Pan Y., Liu H., Yang C., Chen J., Bai B. (2015). Neuroprotective effects of apelin-13 on experimental ischemic stroke through suppression of inflammation. Peptides.

[39] Haghparast E., Sheibani V., Abbasnejad M., Esmaeili-Mahani S. (2019). Apelin-13 attenuates motor impairments and prevents the changes in synaptic plasticity-related molecules in the striatum of Parkinsonism rats. Peptides.

[40] Haghparast E., Esmaeili-Mahani S., Abbasnejad M., Sheibani V. (2018). Apelin-13 ameliorates cognitive impairments in 6-hydroxydopamine-induced substantia nigra lesion in rats. Neuropeptides.

[41] Foroughi K., Khaksari M., Rahmati M., Bitaraf F.S., Shayannia A. (2019). Apelin-13 Protects PC12 Cells Against Methamphetamine-Induced Oxidative Stress, Autophagy and Apoptosis. Neurochem. Res..

[42] Bose A., Beal M.F. (2016). Mitochondrial dysfunction in Parkinson’s disease. J. Neurochem..

[43] Betarbet R., Sherer T.B., MacKenzie G., Garcia-Osuna M., Panov A.V., Greenamyre J.T. (2000). Chronic systemic pesticide exposure reproduces features of Parkinson’s disease. Nat. Neurosci..

[44] Bardag-Gorce F., Francis T., Nan L., Li J., He Lue Y., French B.A., French S.W. (2005). Modifications in P62 occur due to proteasome inhibition in alcoholic liver disease. Life Sci..

[45] Lambert A.J., Brand M.D. (2004). Inhibitors of the quinone-binding site allow rapid superoxide production from mitochondrial NADH:ubiquinone oxidoreductase (complex I). J. Biol. Chem..

[46] Moors T.E., Hoozemans J.J., Ingrassia A., Beccari T., Parnetti L., Chartier-Harlin M.C., van de Berg W.D. (2017). Therapeutic potential of autophagy-enhancing agents in Parkinson’s disease. Mol. Neurodegener..

[47] Anding A.L., Baehrecke E.H. (2017). Cleaning House: Selective Autophagy of Organelles. Dev. Cell.

[48] Minakaki G., Menges S., Kittel A., Emmanouilidou E., Schaeffner I., Barkovits K., Bergmann A., Rockenstein E., Adame A., Marxreiter F. (2018). Autophagy inhibition promotes SNCA/alpha-synuclein release and transfer via extracellular vesicles with a hybrid autophagosome-exosome-like phenotype. Autophagy.

[49] Fowler A.J., Moussa C.E. (2018). Activating Autophagy as a Therapeutic Strategy for Parkinson’s Disease. CNS Drugs.

[50] Xiong N., Xiong J., Jia M., Liu L., Zhang X., Chen Z., Huang J., Zhang Z., Hou L., Luo Z. (2013). The role of autophagy in Parkinson’s disease: Rotenone-based modeling. Behav. Brain Funct. BBF.

[51] Filomeni G., Graziani I., De Zio D., Dini L., Centonze D., Rotilio G., Ciriolo M.R. (2012). Neuroprotection of kaempferol by autophagy in models of rotenone-mediated acute toxicity: Possible implications for Parkinson’s disease. Neurobiol. Aging.

[52] Li S., Pu X.P. (2011). Neuroprotective effect of kaempferol against a 1-methyl-4-phenyl-1,2,3,6-tetrahydropyridine-induced mouse model of Parkinson’s disease. Biol. Pharm. Bull..

[53] Filomeni G., Desideri E., Cardaci S., Graziani I., Piccirillo S., Rotilio G., Ciriolo M.R. (2010). Carcinoma cells activate AMP-activated protein kinase-dependent autophagy as survival response to kaempferol-mediated energetic impairment. Autophagy.

[54] Deng Y.N., Shi J., Liu J., Qu Q.M. (2013). Celastrol protects human neuroblastoma SH-SY5Y cells from rotenone-induced injury through induction of autophagy. Neurochem. Int..

[55] Wu Y., Li X., Zhu J.X., Xie W., Le W., Fan Z., Jankovic J., Pan T. (2011). Resveratrol-activated AMPK/SIRT1/autophagy in cellular models of Parkinson’s disease. Neuro-Signals.

[56] Peng T., Liu X., Wang J., Liu Y., Fu Z., Ma X., Li J., Sun G., Ji Y., Lu J. (2019). Long noncoding RNA HAGLROS regulates apoptosis and autophagy in Parkinson’s disease via regulating miR-100/ATG10 axis and PI3K/Akt/mTOR pathway activation. Artif. Cells Nanomed. Biotechnol..

[57] Bao H.J., Zhang L., Han W.C., Dai D.K. (2015). Apelin-13 attenuates traumatic brain injury-induced damage by suppressing autophagy. Neurochem. Res..

[58] Limanaqi F., Biagioni F., Busceti C.L., Ryskalin L., Polzella M., Frati A., Fornai F. (2019). Phytochemicals Bridging Autophagy Induction and Alpha-Synuclein Degradation in Parkinsonism. Int. J. Mol. Sci..

[59] Parekh P., Sharma N., Gadepalli A., Shahane A., Sharma M., Khairnar A. (2019). A Cleaning Crew: The Pursuit of Autophagy in Parkinson’s Disease. ACS Chem. Neurosci..

[60] Kim S., Kim S., Hwang A.R., Choi H.C., Lee J.Y., Woo C.H. (2020). Apelin-13 Inhibits Methylglyoxal-Induced Unfolded Protein Responses and Endothelial Dysfunction via Regulating AMPK Pathway. Int. J. Mol. Sci..

[61] Yue P., Jin H., Aillaud M., Deng A.C., Azuma J., Asagami T., Kundu R.K., Reaven G.M., Quertermous T., Tsao P.S. (2010). Apelin is necessary for the maintenance of insulin sensitivity. Am. J. Physiol. Endocrinol. Metab..

[62] Alers S., Löffler A.S., Wesselborg S., Stork B. (2012). Role of AMPK-mTOR-Ulk1/2 in the regulation of autophagy: Cross talk, shortcuts, and feedbacks. Mol. Cell. Biol..

[63] Meley D., Bauvy C., Houben-Weerts J.H., Dubbelhuis P.F., Helmond M.T., Codogno P., Meijer A.J. (2006). AMP-activated protein kinase and the regulation of autophagic proteolysis. J. Biol. Chem..

[64] Gross A.S., Zimmermann A., Pendl T., Schroeder S., Schoenlechner H., Knittelfelder O., Lamplmayr L., Santiso A., Aufschnaiter A., Waltenstorfer D. (2019). Acetyl-CoA carboxylase 1-dependent lipogenesis promotes autophagy downstream of AMPK. J. Biol. Chem..

[65] Høyer-Hansen M., Jäättelä M. (2007). AMP-activated protein kinase: A universal regulator of autophagy?. Autophagy.

[66] Laker R.C., Drake J.C., Wilson R.J., Lira V.A., Lewellen B.M., Ryall K.A., Fisher C.C., Zhang M., Saucerman J.J., Goodyear L.J. (2017). Ampk phosphorylation of Ulk1 is required for targeting of mitochondria to lysosomes in exercise-induced mitophagy. Nat. Commun..

[67] Corona Velazquez A.F., Jackson W.T. (2018). So Many Roads: The Multifaceted Regulation of Autophagy Induction. Mol. Cell. Biol..

[68] Yang X., Zhu W., Zhang P., Chen K., Zhao L., Li J., Wei M., Liu M. (2014). Apelin-13 stimulates angiogenesis by promoting cross-talk between AMP-activated protein kinase and Akt signaling in myocardial microvascular endothelial cells. Mol. Med. Rep..

[69] Duan J., Cui J., Yang Z., Guo C., Cao J., Xi M., Weng Y., Yin Y., Wang Y., Wei G. (2019). Neuroprotective effect of Apelin 13 on ischemic stroke by activating AMPK/GSK-3β/Nrf2 signaling. J. Neuroinflamm..

[70] Chen Y., Qiao X., Zhang L., Li X., Liu Q. (2020). Apelin-13 regulates angiotensin II-induced Cx43 downregulation and autophagy via the AMPK/mTOR signaling pathway in HL-1 cells. Physiol. Res..

[71] O’Dowd B.F., Heiber M., Chan A., Heng H.H., Tsui L.C., Kennedy J.L., Shi X., Petronis A., George S.R., Nguyen T. (1993). A human gene that shows identity with the gene encoding the angiotensin receptor is located on chromosome 11. Gene.

[72] Muto J., Shirabe K., Yoshizumi T., Ikegami T., Aishima S., Ishigami K., Yonemitsu Y., Ikeda T., Soejima Y., Maehara Y. (2014). The apelin-APJ system induces tumor arteriogenesis in hepatocellular carcinoma. Anticancer Res..

[73] Picault F.X., Chaves-Almagro C., Projetti F., Prats H., Masri B., Audigier Y. (2014). Tumour co-expression of apelin and its receptor is the basis of an autocrine loop involved in the growth of colon adenocarcinomas. Eur. J. Cancer (Oxf. Engl. 1990).

[74] Lee H.J., Tomioka M., Takaki Y., Masumoto H., Saido T.C. (2000). Molecular cloning and expression of aminopeptidase A isoforms from rat hippocampus. Biochim. Biophys. Acta.

[75] Matsumoto M., Hidaka K., Akiho H., Tada S., Okada M., Yamaguchi T. (1996). Low stringency hybridization study of the dopamine D4 receptor revealed D4-like mRNA distribution of the orphan seven-transmembrane receptor, APJ, in human brain. Neurosci. Lett..

[76] O’Carroll A.M., Selby T.L., Palkovits M., Lolait S.J. (2000). Distribution of mRNA encoding B78/apj, the rat homologue of the human APJ receptor, and its endogenous ligand apelin in brain and peripheral tissues. Biochim. Biophys. Acta.

[77] Xu H.M., Jiang H., Wang J., Luo B., Xie J.X. (2008). Over-expressed human divalent metal transporter 1 is involved in iron accumulation in MES23.5 cells. Neurochem. Int..

[78] Zhang K., Ma Z., Wang J., Xie A., Xie J. (2011). Myricetin attenuated MPP(+)-induced cytotoxicity by anti-oxidation and inhibition of MKK4 and JNK activation in MES23.5 cells. Neuropharmacology.

